# Cytotoxicity of ^212^Pb-labeled anti-PTK7 antibody in 2D adherent and 3D multicellular bladder cancer models

**DOI:** 10.1186/s41181-025-00382-3

**Published:** 2025-08-30

**Authors:** Kim Lindland, Asta Juzeniene

**Affiliations:** 1 Department of Molecular Medicine, Institute of Basic Medical Sciences, University of Oslo, 0316 Oslo, Norway; 2 Department of Radiation Biology, Institute of Cancer Research, The Norwegian Radium Hospital, Oslo University Hospital, 0379 Oslo, Norway; 3 Oncoinvent ASA, 0484 Oslo, Norway; 4 Department of Physics, University of Oslo, 0316 Oslo, Norway

**Keywords:** PTK7 (Protein tyrosine kinase 7), ^212^Pb, Targeted alpha therapy (TAT), Bladder cancer, Monoclonal antibody, Internalization

## Abstract

**Background:**

Bladder cancer remains a significant global health challenge, with approximately 75% of cases presenting as non-muscle-invasive bladder cancer. Despite standard treatment with transurethral resection and intravesical Bacillus Calmette-Guérin immunotherapy, up to 40% of patients develop resistance or progress to muscle-invasive disease. Targeted alpha-emitting radionuclide therapy offers promising therapeutic potential through the selective delivery of high linear energy transfer radiation to tumor cells while minimizing damage to healthy tissues. PTK7 is overexpressed in various malignancies, including bladder cancer, and is therefore a viable therapeutic target. This study evaluated the preclinical efficacy of [^212^Pb]Pb-TCMC-chOI-1, a ^212^Pb-labeled antibody targeting PTK7, for targeted alpha-emitting radionuclide therapy in bladder cancer using 2D adherent cultures (clonogenic assay) and 3D multicellular spheroid models (spheroid growth inhibition).

**Results:**

PTK7 expression analysis revealed varying antigen densities across five bladder cancer cell lines, ranging from approximately 10,000 to 70,000 sites per cell. The chimeric anti-PTK7 antibody demonstrated apparent equilibrium dissociation constants of 10–44 nM with moderate binding affinity suitable for therapeutic applications. [^212^Pb]Pb-TCMC-chOI-1 treatment resulted in activity- and time-dependent cytotoxicity, with enhanced sensitivity observed in cell lines with higher PTK7 levels. In clonogenic assays, the activity concentration required for 50% growth reduction was 48–74 kBq/mL, corresponding to 22–51 bound and 9–16 internalized ^212^Pb atoms per cell. In 3D models, similar therapeutic effects were observed despite significantly lower activities (values of approximately 1 and 30 kBq/mL for KU-19–19 and 647-V cells, respectively), suggesting a more pronounced cross-fire effect. Flow cytometry demonstrated treatment-induced DNA damage, cell cycle perturbations and cell death, with response patterns correlating with overall treatment sensitivity. RT-112 and KU-19–19 cells showed superior responses compared to 647-V and T-24 cells, consistent with their higher PTK7 expression.

**Conclusions:**

These findings support PTK7 as a therapeutic target for bladder cancer and demonstrate the potential of [^212^Pb]Pb-TCMC-chOI-1 for targeted alpha-emitting radionuclide therapy. The results provide a rationale for further preclinical optimization of this therapeutic approach.

**Trial registration number (TRN):** Not applicable.

**Supplementary Information:**

The online version contains supplementary material available at 10.1186/s41181-025-00382-3.

## Background

Bladder cancer (BC) is a major global health burden, ranking as the tenth most commonly diagnosed cancer worldwide (Wéber et al. 2024; Hoogstraten et al. 2023). Non-muscle-invasive bladder cancer (NMIBC) comprises approximately 75% of all newly diagnosed cases (Chu and Porten 2021; Seidl 2020). While transurethral resection followed by intravesical Bacillus Calmette-Guérin (BCG) immunotherapy remains the standard of care for NMIBC, up to 40% of patients develop resistance or experience disease progression to muscle-invasive disease, highlighting the urgent need for novel therapeutic strategies (Hoogstraten et al. 2023; Seidl 2020).In recent years, targeted therapies and immunotherapies have emerged as promising approaches for BC management (Zhang and Ke 2025). These include antibody–drug conjugates (ADCs) and immune checkpoint inhibitors, several of which are under active clinical investigation (Seidl 2020). Among them, targeted alpha-emitting radionuclide therapy (TAT) offers high therapeutic efficacy by delivering potent short-range radiation to tumor cells (Jabbar et al. 2023). Alpha particles, characterized by high linear energy transfer (LET), induce complex and irreparable double-stranded DNA breaks (Pouget and Constanzo 2021). Their short range (< 100 μm) minimizes damage to surrounding healthy tissues (Coll et al. 2023; Sgouros et al. 2020).

The localized nature of NMIBC makes it particularly well-suited for the intravesical delivery of alpha-emitting radiopharmaceuticals. Unlike systemic administration, intravesical instillation directly deposits the therapeutic agent into the bladder lumen, maximizing tumor exposure while minimizing systemic distribution and associated toxicities (Seidl 2020). This route also circumvents the “sink effect” observed with systemic antibody delivery, where circulating antibodies compete for target binding sites, enabling higher effective concentrations at the tumor site (Awwad and Angkawinitwong 2018). In addition, the confined bladder environment allows prolonged contact between the radiopharmaceutical and target tissue, thereby optimizing the therapeutic window for short-lived alpha emitters.

Alpha-emitting radionuclides have been evaluated for TAT for BC, including bismuth-213 (^213^Bi) and astatine-211 (^211^At) (Pfost et al. 2009; Fazel et al. 2015; Autenrieth et al. 2018; Rousseau et al. 2025). These radionuclides have been conjugated to monoclonal antibodies (mAbs) targeting tumor-associated antigens (Jabbar et al. 2023; Pfost et al. 2009; Fazel et al. 2015; Rousseau et al. 2025). Clinical feasibility of TAT-mAb has been demonstrated in early-phase trials (Meredith et al. 2014a, b, 2018). Intravesical application of ^213^Bi-labeled anti-EGFR mAbs in patients with BCG-refractory carcinoma in situ showed promising therapeutic responses with minimal systemic toxicity (Autenrieth et al. 2018). In addition, ^211^At-labeled anti-CA-IX antibody have been explored in preclinical models of NMIBC for intravesical TAT (Rousseau et al. 2025). High binding affinity and potent cytotoxicity of [^211^At]At-anti-CA-IX were observed in RT-112 BC cells, with superior efficacy compared to a beta-emitting analog (Rousseau et al. 2025). Biodistribution and toxicity studies in mice indicated low systemic uptake and no bladder abnormalities, and clinical feasibility was demonstrated by PET/CT imaging in NMIBC patients, supporting the safety and potential of this approach for future clinical trials (Rousseau et al. 2025). These studies support the potential of TAT as a bladder-sparing therapy (Pfost et al. 2009; Fazel et al. 2015; Autenrieth et al. 2018; Rousseau et al. 2025).

Lead-212 is a beta emitter that serves as an in vivo generator of alpha-emitting daughters, notably ^212^Bi, which decays further to emit high-LET alpha particles (Kokov et al. 2022). It is emerging as a promising radionuclide for TAT, owing to its suitable half-life (10.6 h) and established chelation chemistry (Baidoo et al. 2013). Production from thorium-228 (^228^Th) based generators enables a scalable and high-purity supply, supporting broader clinical applications (Kokov et al. 2022). When conjugated to monoclonal antibodies or peptides, selective targeting of tumor-associated antigens is enabled by the ligand, while ^212^Pb provides the cytotoxic payload, supporting application across a broad range of malignancies (Kokov et al. 2022; Yong and Brechbiel 2011). Several ^212^Pb-labeled conjugates are currently undergoing evaluation in both preclinical models and early-phase clinical trials, reflecting growing interest in its therapeutic potential (Plichta and Buatti 2025; Zimmermann 2024).

Protein Tyrosine Kinase 7 (PTK7) is a pseudokinase involved mainly in non-canonical Wnt signaling, regulating processes such as cell migration and proliferation (Dessaux et al. 2024). PTK7 is overexpressed in various cancers, including BC (Jin et al. 2024). It has been explored as a target for ADCs, aptamer-drug conjugates, diagnostic imaging and prognostic biomarker (Dessaux et al. 2024; Xiang et al. 2022; Mottard et al. 2024; Cho et al. 2025). For instance, a PTK7 aptamer-gemcitabine conjugate (PTK7-GEMs) demonstrated tumor-selective uptake and cytotoxicity with limited off-target effects in preclinical BC models (Xiang et al. 2022). Ongoing clinical trials are investigating PTK7 as a diagnostic and prognostic marker for BC (NCT06005116).

In our previous study, we administered intraperitoneal [^212^Pb]Pb-TCMC-chOI-1 in an ovarian cancer model, achieving significant tumor regression and demonstrating potent targeted cytotoxicity (Lindland et al. 2025). Based on these results, we assessed [^212^Pb]Pb-TCMC-chOI-1, a ^212^Pb-labeled chimeric anti-PTK7 mAb, across a panel of BC models. This study evaluated PTK7 expression, antibody binding affinity, internalization kinetics, and cytotoxic effects in 2D and 3D multicellular spheroid models in vitro. To our knowledge, this is the first report evaluating PTK7-TAT using ^212^Pb in BC.

## Material and methods

### Antibodies

The chimeric anti-PTK7 monoclonal IgG1 antibody, designated chOI-1, was developed and produced as described previously (Lindland et al. 2025). Ultra-LEAF purified human IgG1 isotype control (clone QA16A12) was acquired from Nordic Biosite (Kristiansand, Norway). Both antibodies were labeled with Alexa-Fluor488 (Alexa488) using a Protein Labeling Kit (Thermo Fisher Scientific, A10235, Oslo, Norway) following the manufacturer's instructions.

### Cell lines

Human BC cell lines CAL-29, RT-112, KU-19-19, 647-V, and T-24 were purchased from the Leibniz Institute DSMZ (Braunschweig, Germany), and the osteosarcoma cell line OHS was established at the Norwegian Radium Hospital (Fodstad et al. 1986). CAL-29, T-24, and 647-V cells were cultured in DMEM (Life Technologies Invitrogen, Thermo Scientific, Waltham, MA, USA), while OHS, KU-19–19, and RT-112 cells were cultured in RPMI-1640 medium (Life Technologies Invitrogen, Thermo Scientific, Waltham, MA, USA). The medium was supplemented with 10% heat-inactivated fetal bovine serum (FBS) and 1% penicillin–streptomycin. Cells were maintained in an incubator at 37 °C and 5% CO_2_. At 80–90% confluence, the cells were harvested using TrypLE Express (Fisher Scientific, Oslo, Norway).

### Preparation of ^212^Pb and radioactivity measurements

Lead-212 was generated from ^228^Th (Eckert & Ziegler, Braunschweig, Germany) via radon-220 (^220^Rn) emanation using a single-chamber glass flask system, as previously described (Li et al. 2023). Briefly, ^228^Th in 1 M HCl was applied to quartz wool in the cap of an inverted 100 mL flask, allowing ^220^Rn to emanate and deposit ^212^Pb on the inner surface of the flask. After 1–2 days, ^212^Pb was collected by rinsing with 0.1 M HCl, and its activity was quantified using a Cobra II Auto-gamma Counter (Packard Instrument Company, Downer Grove, Illinois, USA) set to a 50–120 keV counting window or a Capintec CRC-25R radioisotope dose calibrator (Capintec Inc., Ramsey, NJ, US) with a dial setting of 667 (Bergeron et al. 2022; Napoli et al. 2020).

### Radiolabeling and quality control of radioimmunoconjugates

Both antibodies were conjugated with the bifunctional chelator S-2-(4-isothiocyanatobenzyl)-1,4,7,10-tetraaza-1,4,7,10-tetra(2-carbamoylmethyl)cyclododecane (p-SCN-Bn-TCMC, TCMC; Macrocyclics Inc., Dallas, USA) as previously described (Lindland et al. 2025). The extracted ^212^Pb was adjusted to a pH of 5–6 using 5 M sodium acetate (Merck, Darmstadt, Germany) and combined with TCMC-mAbs at specific activities ranging from 5 to 50 MBq/mg for 30–35 min at 37 °C. The resulting [^212^Pb]Pb-TCMC-mAb (refers to both antibodies conjugated and radiolabeled with ^212^Pb, i.e. [^212^Pb]Pb-TCMC-hIgG and [^212^Pb]Pb-TCMC-chOI-1) was formulated in Dulbecco’s phosphate-buffered saline (Sigma-Aldrich, Oslo, Norway) containing 7.5% recombinant albumin (Octapharma, Lachen, Switzerland), 200 mM sodium ascorbate (Sigma-Aldrich, Oslo, Norway), and 1 mM EDTA (pH 7.4; (Sigma-Aldrich, Oslo, Norway). Radiochemical purity was evaluated using instant thin-layer chromatography strips (Biodex, Shirley, NY, USA), as described previously (Lindland et al. 2025). Radioimmunoconjugates (RICs) with radiochemical purity > 95% were used in the experiments.

The immunoreactive fraction (IRF) was determined using one-point binding assays with cultured bladder cancer cell lines and frozen stocks of OHS cells, which were highly positive for PTK7 antigen (Lindland et al. 2025). Non-specific binding was controlled by pre-incubation with excess unlabeled chOI-1, following an established protocol (Lindland et al. 2025).

### Measurement of PTK7 expression on bladder cancer cells

PTK7 surface expression was quantified by flow cytometry using saturation binding of Alexa488-labeled chOI-1, as previously described for ovarian cancer cells [28]. Briefly, cells in suspension were incubated with increasing concentrations of Alexa488-chOI-1 (1–100 nM) at 4 °C, washed, and stained with MitoTracker Red CMXRos and DRAQ5 to discriminate live/dead cells. Data analysis and calculation of relative median fluorescence intensity (rMFI) and apparent equilibrium dissociation constant (K_D_^APP^) followed the established methodology (Lindland et al. 2025).

Saturation binding with [^212^Pb]Pb-TCMC-chOI-1 (1–100 nM) was performed analogously, with non-specific binding assessed using excess unlabeled antibody and radioactivity measured in the cell pellet. The number of specifically bound molecules per cell and B_max_ were determined as described previously (Lindland et al. 2025). All data were fitted to a one-site binding model using GraphPad Prism.

### Binding and internalization of [^212^Pb]Pb-TCMC-mAb

The assessment of [^212^Pb]Pb-TCMC-mAb binding and internalization was performed as described in our earlier work (Lindland et al. 2024), with modifications in activity concentrations, time points, and cell models. The total and internalized activities were quantified in 2D monolayer cultures. BC cells were incubated with [^212^Pb]Pb-TCMC-mAb at 10 kBq/mL (specific activity 50 MBq/mg), and binding/internalization was evaluated at 1, 4, and 24 h, as well as at increasing activity concentrations at 4 h (20–80 kBq/mL). Surface-bound antibodies were removed using trypsin-based stripping buffer (0.01 g/mL in PBS, pH 8–9, 1 h, 37 °C), and radioactivity in both the stripping buffer (surface) and cell pellet (internalized) was measured (Lindland et al. 2024). The percentage of internalized radioactivity was calculated as (internalized activity/total activity) × 100. The number of 212Pb atoms per cell was calculated based on the formula *A* = λ*N*, where *A* is the activity in becquerels, λ is the decay constant, and *N* is the number of atoms.

### Cytotoxic effects of [^212^Pb]Pb-TCMC-mAbs in clonogenic assay

The cytotoxicity of [^212^Pb]Pb-TCMC-mAbs was assessed using a clonogenic assay T-24, KU-19–19, 647-V, and RT-112 cells. Attempts with CAL-29 cells were unsuccessful because they failed to form colonies. Cells were seeded in T25 flasks and allowed to adhere overnight. The next day, the cells were treated with [^212^Pb]Pb-TCMC-mAbs (5 or 50 MBq/mg, 0–80 kBq/mL) for 1, 4, or 24 h at 37 °C. The untreated controls were maintained in the culture medium. After treatment, the radioactive medium was replaced with fresh culture medium, and the cells were incubated under standard conditions (37 °C, 5% CO_2_) until colonies containing at least 50 cells were formed. Colony formation required approximately 7–10 days for all the tested cell lines.

Colonies were fixed with 96% ethanol and stained with 0.4% methylene blue (Thermo Fisher Scientific, Waltham, MA, USA). Colonies were counted manually, and the clonogenic fractions were calculated relative to the plating efficiency of the untreated controls. All experiments were conducted using three biological replicates and three technical replicates to ensure reproducibility.

### Cytotoxic effects of [^212^Pb]Pb-TCMC-mAbs in 3D multicellular models

3D multicellular spheroids were generated from two BC cell lines, KU-19–19 and 647-V, using the liquid overlay technique (Carlsson and Yuhas 1984; Ivascu and Kubbies 2006). Briefly, flat-bottom 96-well plates were coated with a 1.5% (w/v) agarose (Sigma-Aldrich, Oslo, Norway) solution in PBS containing Ca^2⁺^ and Mg^2⁺^ to prevent cell adhesion. A total of 500 cells in 100 µL of culture medium were seeded in agarose-coated plates per well. The plates were centrifuged at 1500 rpm for 15 min to facilitate spheroid formation and incubated under standard conditions (37 °C, 5% CO_2_). Spheroids were formed within four days of seeding. Spheroid formation was unsuccessful in CAL-29, T-24, and RT-112 cells, as they did not exhibit proper growth curves suitable for reliable treatment effect evaluation over the 14-day study period (Fig. S1).

On day 0 (4 days after seeding the cells), spheroids were treated with increasing concentrations of [^212^Pb]Pb-TCMC-mAb (0–50 kBq/mL, 5 or 50 MBq/mg) for 1, 4, or 24 h. To assess the specificity of binding, a subset of spheroids was pre-incubated with 20 µg unlabeled chOI-1 for 30 min before RIC treatment. Untreated controls were maintained in the cell medium. After incubation with RIC, the spheroids were washed six times with fresh medium to remove unbound RIC and further incubated under standard conditions for up to 14 days. Spheroid growth was monitored by bright-field imaging on days 0, 3, 7, 10, and 14 using a Carl Zeiss Axiovert inverted fluorescence microscope (× 4 objective; Carl Zeiss AG, Baden-Württemberg, Germany), and the culture medium was replaced immediately after each imaging session.

On day 14, the spheroids were stained with fluorescein diacetate (FDA; Sigma-Aldrich) and propidium iodide (PI; Sigma-Aldrich) to distinguish between live and dead cells. FDA was used to assess cell viability by detecting enzymatic activity in live cells, whereas PI was used to identify dead cells with compromised membrane integrity. The stained spheroids were imaged using the microscope setup described above. Image analysis was performed using AxioVision Rel. version 4.8 software (Carl Zeiss AG), and representative images from a single experiment were provided.

The doubling times of the spheroids were calculated using GraphPad Prism 10. An exponential growth model was applied to the data, and the growth curve was fitted to the equation y = ae^bx^, where *y* represents the spheroid size, *a* is the initial size, *b* is the exponential growth rate constant, and *x* is time. Time values were entered as independent variables (X) and spheroid size as dependent variables (Y). Nonlinear regression was performed using the “Exponential Growth” equation from the growth equation panel. The parameter *b* was estimated using the software, and the doubling time (DT) was calculated as ln(2)/b.

The half-maximal inhibitory activity concentrations (IC₅₀) values for spheroid growth inhibition were estimated by plotting the normalized final spheroid size (treated/control) against the activity concentration and identifying the concentration at which spheroid growth was reduced by 50% compared to the untreated controls. With only three concentrations tested, the IC_50_ was approximated by interpolation between the two concentrations that bracketed the 50% inhibition point.

### Assessment of cell viability, cell cycle progression, and DNA damage

Flow cytometry-based analyses were conducted to evaluate cell viability, cell cycle progression, and DNA damage in two BC cell lines, KU-19-19 and 647-V. Cells (0.2–0.6 × 10^6^) were seeded in T25 or T75 flasks and incubated overnight under standard culture conditions. The following day, the cells were treated with 100 kBq/mL [^212^Pb]Pb-TCMC-chOI-1 for 24 h at 37 °C. The control cells were left in the cell medium. After treatment, the medium containing RIC was replaced with fresh culture medium, and the cells were harvested for analysis on days 1 (immediately after treatment removal), 3, and 6 post-treatment.

To evaluate cell viability, the cells were stained with Annexin V-FITC (ImmunoTools, Friesoythe, Germany) and propidium iodide (PI; 1.9 µg/mL; Sigma-Aldrich, Oslo Norway) in Annexin binding buffer (10 mM HEPES, 140 mM NaCl, and 2.5 mM CaCl_2_) for 10–15 min at room temperature.

For cell cycle distribution and DNA damage analysis, cells were stained with fixable viability dye eFluor450 (Invitrogen, Thermo Fisher Scientific) on ice for 60 min to distinguish live cells from dead cells. Following staining, the cells were fixed in 100% methanol (Sigma-Aldrich, Oslo Norway) and washed with PBS without Mg^2+^ and Ca^2+^ containing 0.2% (v/v) Tween-20 (PBST; Sigma-Aldrich Oslo, Norway). The fixed cells were then incubated with primary antibodies against phosphorylated histone H2AX (pγH2AX; Phospho-Histone H2A.X (Ser139) mAb #CR55T33, eBioscience™, Invitrogen™, Fisher Scientific; lot: 3,040,381) at a concentration of 1 µg/mL for DNA damage detection and phosphorylated histone H3 (anti-pS10H3; rabbit mAb #06–570; Merck Millipore) for mitotic tracking along with RNase A (0.4 mg/mL; PureLink, Thermo Fisher Scientific) in PBST supplemented with 2% (v/v) FBS for 60 min at room temperature in the dark. After washing with PBST, the cells were incubated with secondary antibodies: donkey anti-rabbit AlexaFluor 647-conjugated (#A-31573; Invitrogen, Thermo Fisher Scientific) for anti-pS10H3 and goat anti-mouse FITC-conjugated (#F0479; Agilent Technologies, Santa Clara, CA, USA) for pγH2AX at a concentration of 2 µg/mL in PBST supplemented with 2% (v/v) FBS for 30 min at room temperature in the dark. Finally, the samples were stained with PI (10 µg/mL) in PBST.

Data acquisition was performed using a CytoFlex S Flow Cytometer (Beckman Coulter), and all data were analyzed using the CytExpert software version 2.4.

### Statistical analysis

Data are presented as mean ± standard deviation (SD) from at least three independent experiments. Given the small sample sizes (n = 3–4), we first evaluated the data distribution using the Shapiro–Wilk normality test. For ANOVA, homogeneity of variances was confirmed using Bartlett’s test. Because these assumptions were met, parametric methods were applied. Differences among groups were determined using one-way ANOVA with Holm-Sidak correction for multiple comparisons, and pairwise differences were evaluated using multiple unpaired t-tests with Holm-Sidak correction to account for multiple comparisons. Statistical significance was set at *p* < 0.05. GraphPad Prism version 10.4.1 (GraphPad Software, La Jolla, CA, USA) was used for all statistical analyses and curve-fitting. SigmaPlot 15.0 software (Systat Software, Inc., San Jose, CA, USA) was used for Pearson’s correlation analysis.

## Results

### PTK7 expression and binding of chOI-1 to BC cell lines

The binding affinity and antigen density of chOI-1 antibody were evaluated in five BC cell lines (Table 1). K_D_^APP^ values were determined by flow cytometry saturation experiments using AlexaFluor488-labeled chOI-1 and a radiolabeled saturation binding assay with [^212^Pb]Pb-TCMC-chOI-1 (Table 1, Fig. S2). Flow cytometry revealed varying PTK7 expression levels across BC cell lines (Fig. S2). CAL-29 cells showed the highest binding affinity (K_D_^APP^ = 10.4 ± 1.6 nM), followed by RT-112, KU-19–19, T-24, and 647-V cells (Table 1), respectively. The K_D_^APP^ values derived from the [^212^Pb]Pb-TCMC-chOI-1 saturation assay were consistently higher across all cell lines, except for T-24 (Table 1). Despite these differences, both methods demonstrated consistent trends in relative binding affinities across the cell lines.

**Table 1:** Binding characteristics, antigen density, and immunoreactive fraction of chOI-1 in bladder cancer cell lines

Cell line	K_D_^APP^ ± SD (n = 3)* nM	K_D_^APP^ ± SD (n = 3–4)** nM	Antigen sites per cell ± SD (n = 3–4)**	Antigen sites per cell adjusted for internalization**	IRF ± SD (%, n = 3–4)
CAL-29	10.4 ± 1.6	26.2 ± 7.2	76841 ± 6757	69772	26.0 ± 2.0
RT-112	13.7 ± 3.1	44.3 ± 12.1	61416 ± 7052	48897	24.0 ± 7.6
KU-19-19	17.1 ± 2.2	36.9 ± 5.9	53443 ± 3296	36181	22.0 ± 4.0
647-V	19.9 ± 6.4	37.0 ± 9.3	43737 ± 4263	38576	22.0 ± 2.0
T-24	17.8 ± 3.3	15.1 ± 8.7	14629 ± 2192	11030	12.0 ± 3.9

*Flow cytometry saturation

The table summarizes the apparent equilibrium dissociation constants (K_D_^APP^) for chOI-1 binding determined by flow cytometry, the [^212^Pb]Pb-TCMC-chOI-1 saturation assay, antigen sites per cell (adjusted and unadjusted for internalization), and immunoreactive fraction (IRF) for BC cell lines. K_D_^APP^ values were calculated using a one-site binding model. The IRF was determined using one-point binding assays to assess the fraction of radiolabeled antibodies capable of specific binding. Data are presented as mean ± standard deviation (SD) from three to four biological experiments

**[^212^Pb]Pb-TCMC-chOI-1 saturation assay

Antigen sites per cell were quantified using a [^212^Pb]Pb-TCMC-chOI-1 saturation assay, and the values were adjusted to account for receptor internalization based on the experimentally determined internalization after 1 h (Table S1). Quantitative analysis estimated PTK7 densities ranging from approximately 1 × 10^4^ to 7 × 10^4^ sites per cell. CAL-29 cells had the highest antigen density (69 772 sites per cell), followed by RT-112, KU-19–19, 647-V, and T-24 cells (Table 1).

The IRF for OHS cells was determined to be 44.3 ± 4.6% (n = 12), indicating moderate immunoreactivity of the radiolabeled [^212^Pb]Pb-TCMC-chOI-1 and confirming its suitability for use in subsequent experiments. Across all BC cell lines, the IRF values ranged from 12.0 ± 3.9% (T-24) to 26.0 ± 2.0% (CAL-29) (Table 1). The highest IRF values correlated with cell lines exhibiting higher antigen density and stronger binding affinity (CAL-29 and RT-112).

### Surface bound and internalized [^212^Pb]Pb-TCMC-chOI-1 in BC cell lines

**Fig. 1:**
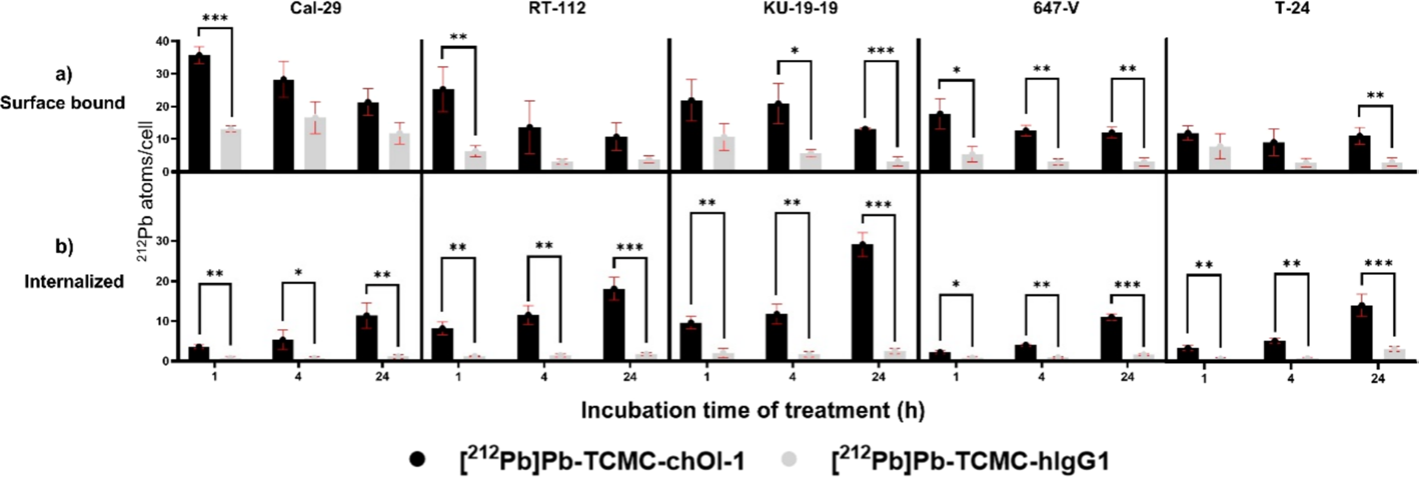
Binding and internalization of [^212^Pb]Pb-TCMC-chOI-1 and [^212^Pb]Pb-TCMC-hlgG in bladder cancer cells over time. Cells were incubated with 10 kBq/ml [^212^Pb]Pb-TCMC-chOI-1 or [^212^Pb]Pb-TCMC-hIgG1 (both at 50 MBq/mg) for 1, 4, and 24 h at 37 °C. After incubation, the cells were washed and treated with a stripping buffer containing trypsin for 1 h at 37 °C to remove the surface-bound ^212^Pb-TCMC-mAbs. The soluble fraction (cell-surface-bound) and cell pellet (internalized) were collected after additional washes were performed. Cell surface-bound and internalized activities were measured using the gamma counter. A) Surface-bound and B) internalized ^212^Pb atoms per cell. The results are presented as the average of three to four biological experiments with error bars. P-values were calculated using an unpaired t-test with correction for multiple comparisons using the Holm-Sidak method (*p < 0.05, **p < 0.005, ***p < 0.0005)

The surface binding and internalization kinetics of [^212^Pb]Pb-TCMC-chOI-1 were quantified in BC cell lines using [^212^Pb]Pb-TCMC-hIgG as an isotype control to distinguish specific PTK7-mediated uptake from non-specific interactions. Measurements were performed at 1, 4, and 24 h, reporting both the number of surface-bound and internalized atoms per cell and the percentage of internalization (Table S1, Fig. 1).

For [^212^Pb]Pb-TCMC-chOI-1, CAL-29 cells exhibited the highest initial surface binding (36 atoms/cell at 1 h), which decreased to 20 atoms/cell at 24 h, while internalized atoms increased from 3 to 10 per cell. The percentage of internalization increased from 8% at 1 h to 33% at 24 h, indicating gradual uptake. RT-112 and KU-19–19 cells showed higher internalization efficiencies, reaching 62% and 68% internalization at 24 h, respectively. KU-19–19 cells demonstrated rapid uptake, with internalized atoms increasing from 8 to 28 atoms per cell at 1 and 24 h respectively. In contrast, T-24 and 647-V cells had lower internalization (24–47% at 24 h), consistent with their lower antigen densities.

Across all cell lines, the isotype control showed lower binding and minimal internalization (surface-bound: 3–17 atoms/cell; internalized: 1–3 atoms/cell; internalization: 5–46%), confirming the specificity of chOI-1 for PTK7.

Two temporal phases were observed: from 1–4 h, only a fraction of the decrease in surface-bound activity was explained by internalization (25–66%), suggesting additional loss mechanisms (e.g., dissociation or degradation); from 4–24 h, internalization accounted for most of the surface-bound loss. At 24 h, the ranking by internalization percentage was KU-19–19 > RT-112 > T-24 > 647-V > CAL-29, paralleling the absolute number of internalized atoms per cell.

These data establish that [^212^Pb]Pb-TCMC-chOI-1 binding and internalization are cell line-dependent, closely reflecting PTK7 expression and internalization capacity. The isotype control confirmed the specificity.

### Cytotoxicity of [^212^Pb]Pb-TCMC-chOI-1 in 2D adherent cells

**Fig. 2:**
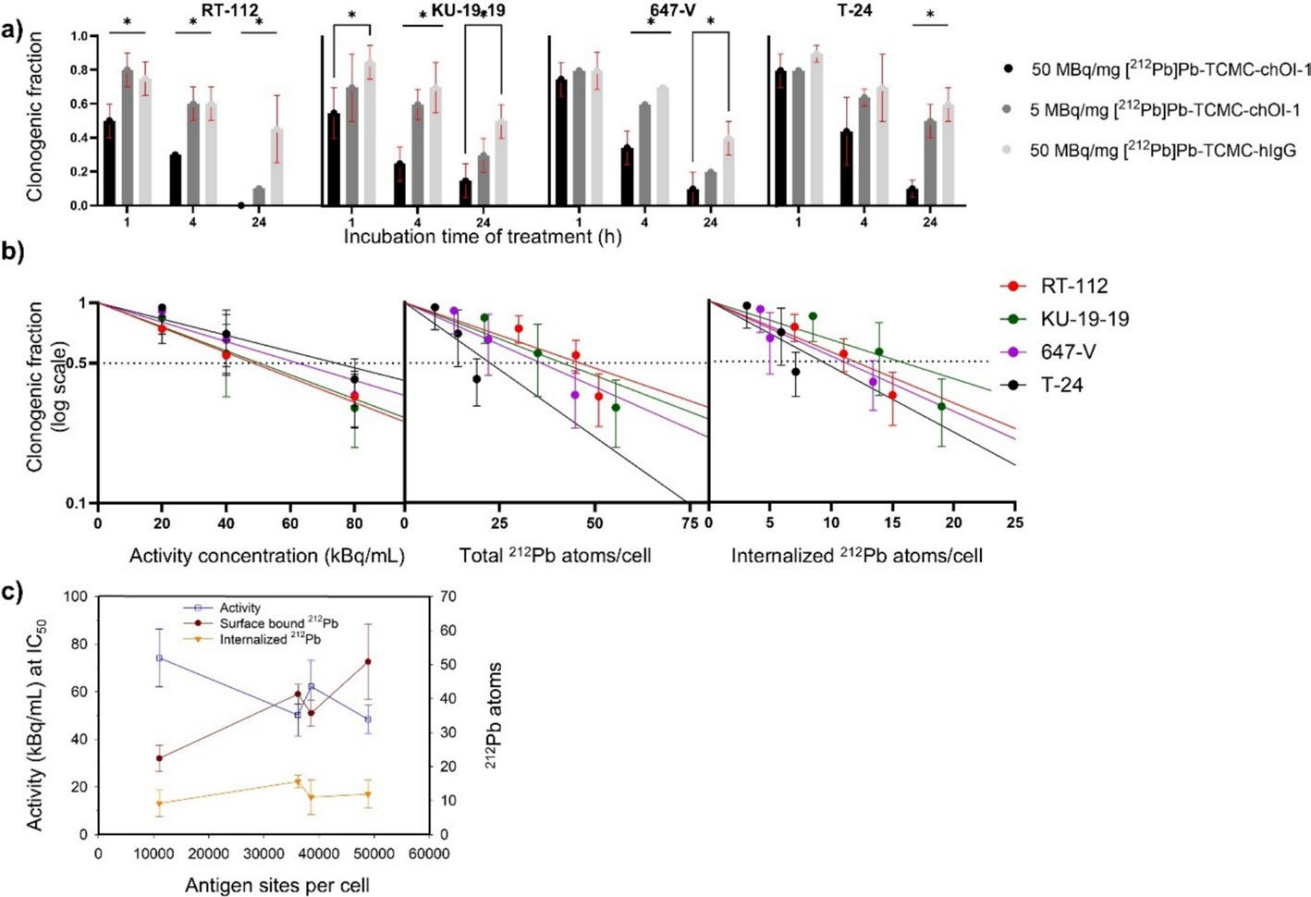
Clonogenic fractions of bladder cancer cell lines treated with [^212^Pb]Pb-TCMC-chOI-1. **A**) Clonogenic fractions of RT-112, KU-19–19, 647-V, and T-24 cells treated with 80 kBq/mL [^212^Pb]Pb-TCMC-chOI-1 of varying specific activities (5 MBq/mg or 50 MBq/mg) or isotype control ([^212^Pb]Pb-TCMC-hIgG) across incubation times of 1, 4, and 24 h; The black line above the bars (**—)** compares 50 MBq/mg with both 5 MBq/mg and the isotype control group. **B**) Clonogenic fractions as a function of activity concentration (0–80 kBq/mL), total, or internalized ^212^Pb atoms/cell after a fixed incubation time of 4 h fitted using the single-hit model (Clonogenic fraction = exp(− A/A_0_), where A is the activity (kBq/mL), and A_0_ is the activity to reduce survival by 67%. **C**) The IC_50_ values of activity concentration, and surface bound and internalized ^212^Pb atoms per cell were plotted against the number of antigen sites per cell. Statistical significance was determined using an unpaired t-test with Holm-Sidak correction (*p < 0.05). Results are presented as mean ± SD from three independent experiments

Clonogenic assays revealed a clear activity- and time-dependent loss of reproductive capacity in all four BC lines after exposure to [^212^Pb]Pb-TCMC-chOI-1. At the highest specific activity (50 MBq/mg), surviving fractions were already reduced after 1 h of incubation and fell to < 10% in RT-112 and KU-19–19 and < 20% in 647-V and T-24 after 24 h, whereas the 5 MBq/mg preparation or the [^212^Pb]Pb-TCMC-hIgG produced only modest effects over the same interval (Fig. 2A). Fitting survival data at 4 h to a single-hit model, IC₅₀ values ranged from 48.4 ± 5.9 kBq/mL (RT-112, most sensitive) to 74.2 ± 12.1 kBq/mL (T-24, least sensitive), with intermediate values for KU-19–19 and 647-V (50 and 62 kBq/mL, respectively; Fig. 2B and Table S2). The number of internalized ^212^Pb atoms per cell required for 50% clonogenic reduction was relatively consistent across cell lines (9–16 atoms/cell corresponding to ~ 13000–23000 antibodies/cell; Table S2), despite differences in overall activity required. While these atomic numbers provide a basis for therapeutic effect correlation, detailed subcellular dosimetric modeling would be required to accurately quantify absorbed doses delivered to critical cellular targets and to establish precise dose–response relationships for clinical translation.

To assess whether the differences in uptake and efficacy parameters were statistically significant across cell lines, we performed a one-way ANOVA. The administered activity and surface-bound ^212^Pb levels differed significantly among the cell lines (*p* = 0.0396 and *p* = 0.0039, respectively), indicating that RIC binding varies among cells. No statistically significant differences were observed in the internalized atoms (*p* = 0.3232), although variability was observed.

To further explore the mechanistic relationships, Pearson’s correlation analysis was performed (Fig. 2C, Table S3). A strong and significant correlation was observed between PTK7 antigen density and surface-bound atoms (r = 0.953, *p* = 0.0474), indicating that cell surface binding scales with antigen levels. A strong inverse and significant correlation was found between surface-bound atoms and administered activity (r = − 0.960, *p* = 0.0399), suggesting that greater radioligand binding reduces the required activity for tumor uptake. Internalized atoms showed moderate correlations with both antigen expression (r = 0.508) and bound atoms (r = 0.588), but these were not statistically significant. Similarly, the correlation between internalized atoms and administered activity (r = − 0.789) showed a strong trend but did not reach significance (*p* = 0.211).

### Cytotoxicity of [^212^Pb]Pb-TCMC-chOI-1 in 3D multicellular models

**Fig. 3:**
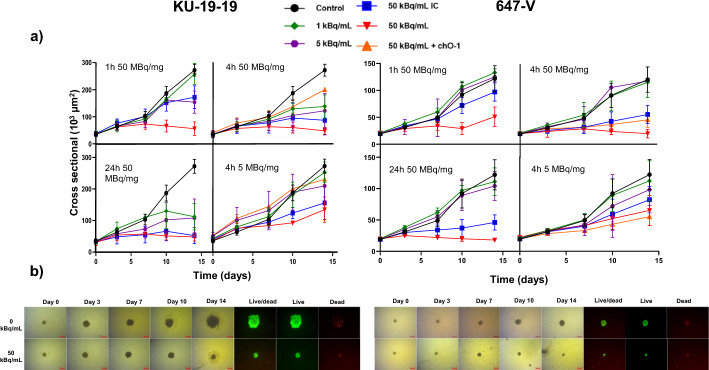
Growth inhibition of bladder cancer spheroids treated with [^212^Pb]Pb-TCMC-mAbs. **A** Growth curves showing spheroid size over time for KU-19–19 (left) and 647-V (right) spheroids treated with radiolabeled [^212^Pb]Pb-TCMC-chOI-1 or [^212^Pb]Pb-TCMC-hIgG (isotype control = IC, blue line) at different activity concentrations for incubation periods of 1, 4, and 24 h. The orange line indicates pre-treatment with chOI-1 for 30 min. The control consisted of spheroids maintained in the cell medium. **B** Representative brightfield and fluorescent images of KU-19-19 and 647-V spheroids treated with control or 50 kBq/mL radiolabeled antibody for 24 h. Spheroids were stained with fluorescein diacetate (FDA; green channel for live cells) and propidium iodide (PI; red channel for dead cells). The merged images show the live/dead cell distributions within the spheroids over time. Scale bars = 200 µm

In 3D spheroid models (KU-19-19 and 647-V), [^212^Pb]Pb-TCMC-chOI-1 inhibited growth in an activity concentration-, time-, and specific-activity-dependent manner (Fig. 3A). At the start of treatment (day 0), the spheroids in both cell lines were approximately 150–200 μm in diameter. Untreated spheroids doubled in size in approximately 5 days (Table S4). KU-19-19 spheroids showed a modest growth delay at 1 kBq/mL and complete inhibition at 50 kBq/mL across all incubation times (50 MBq/mg). Pre-incubation with unlabeled chOI-1 reduced efficacy, and [^212^Pb]Pb-TCMC-hIgG showed moderately delayed tumor growth. 647-V spheroids were less sensitive, with complete inhibition only at 50 kBq/mL after prolonged exposure; lower concentrations produced delayed but measurable growth. Higher specific activity and longer exposure consistently enhanced growth inhibition in both cell lines. Estimated IC₅₀ values were ~ 1 kBq/mL for KU-19–19 and ~ 30 kBq/mL for 647-V (Fig. S3, Table S2). Live/dead staining at day 14 confirmed that high activity suppressed proliferation but did not eradicate all cells (Fig. 3B).

### Comparison of IC_50_ in 2D clonogenic and 3D spheroid assays

Comparison revealed greater sensitivity in 3D spheroids than in 2D monolayers: for KU-19-19, IC_50_ was ~ 1 kBq/mL in 3D versus 50 kBq/mL in 2D; for 647-V, ~ 30 kBq/mL in 3D versus 62 kBq/mL in 2D (Fig. S3, Table S2) after 4 h of treatment. This pattern of increased sensitivity in 3D spheroids compared to 2D monolayers was also consistent following both 1 h and 24 h treatments. This suggests the enhanced efficacy of [^212^Pb]Pb-TCMC-chOI-1 in 3D tumor-like structures. However, precision of 3D IC_50_ values is limited by the number of concentrations tested.

### Assessment of cell viability, cell cycle progression, and DNA damage

**Fig. 4:**
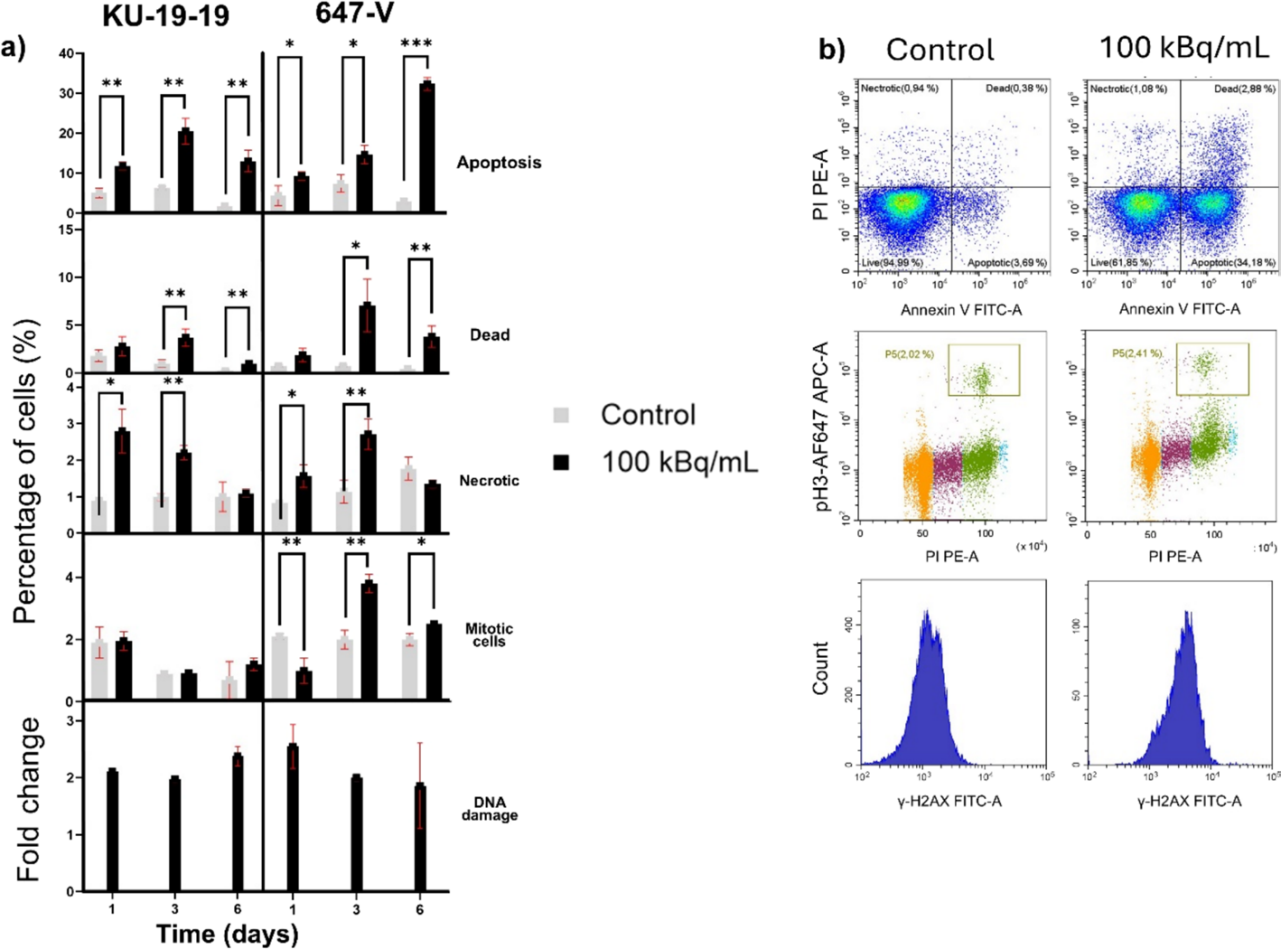
Cell viability, apoptosis, DNA damage, and mitotic analysis in KU-19-19 and 647-V cells following [^212^Pb]Pb-TCMC-chOI-1 treatment. **A** Quantification of apoptotic, necrotic, dead, mitotic, and γH2AX-positive (DNA damage) fractions in KU-19-19 and 647-V BC cells after exposure to 100 kBq/mL [^212^Pb]Pb-TCMC-chOI-1 for 24 h. Control refers to cells left in the cell medium. For apoptosis and cell death analysis, cells were harvested on days 1, 3, and 6 post-treatment, stained with Annexin V-FITC and propidium iodide, and analyzed using flow cytometry (CytoFLEX S, CytExpert software). To assess mitotic cells and DNA double-strand breaks, collected cells were stained with FVD-eFluor450, anti-pS10H3/AlexaFluor 647 (mitosis), anti-γH2AX/FITC (DNA damage), and propidium iodide for the flow analysis. All fractions are presented as mean ± SD from three independent experiments performed in triplicates. DNA damage is presented as fold change compared to that in control cells. Statistical significance was determined using an unpaired t-test with Holm-Sidak correction for multiple comparisons (*p < 0.05, **p < 0.005, ***p < 0.0005). **B** Representative flow cytometry plots showing untreated 647-V cells (left) and cells treated with 100 kBq/mL of [212Pb]Pb-TCMC-chOI-1 (right) on day 6 after treatment

Treatment with 100 kBq/mL [^212^Pb]Pb-TCMC-chOI-1 for 24 h induced significant cytotoxic effects in 2D models of KU-19-19 and 647-V BC cells, as assessed by flow cytometry (Fig. 4). Compared to untreated controls, [^212^Pb]Pb-TCMC-chOI-1 significantly increased cell death. Apoptotic cells exceeded 20% at peak time points, whereas necrotic cells remained below 3%, indicating that apoptosis was the dominant mechanism of cell death. In KU-19-19 cells, apoptosis peaked at day 3 post-treatment, whereas 647-V cells exhibited a slower, cumulative increase in cell death, with maximal apoptotic levels observed on day 6 (Fig. 4).

Mitotic activity remained unchanged in KU-19–19 cells but showed a transient decrease at day 1, followed by recovery in 647-V cells (Fig. 4).

Quantification of γH2AX levels indicated a persistent twofold increase in DNA double-strand breaks in both cell lines compared to the cells left in the medium throughout the 6-day observation period (Fig. 4).

**Fig. 5:**
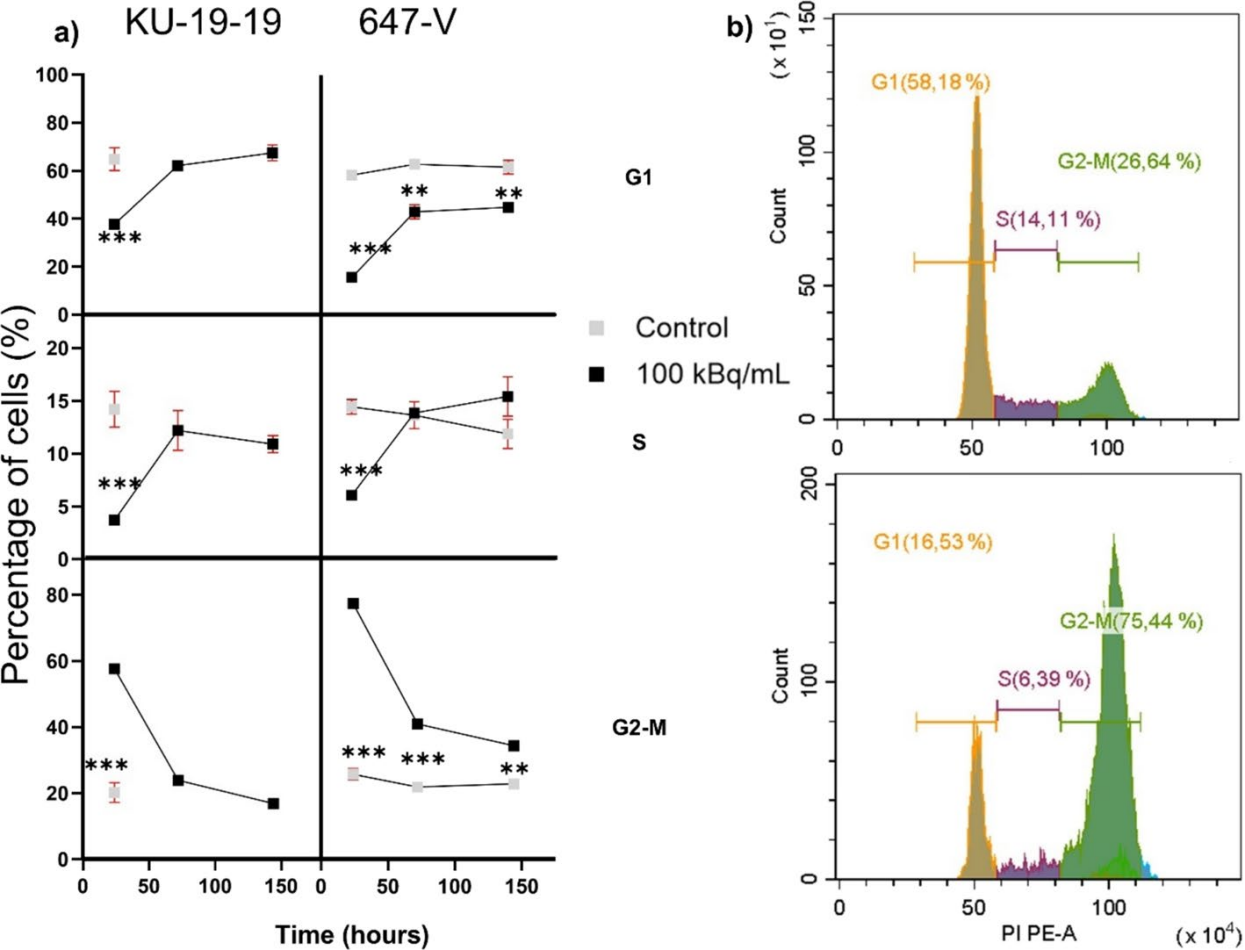
Cell cycle progression of KU-19-19 and 647-V after treatment with [^212^Pb]Pb-TCMC-chOI-1 **A** Cell cycle distribution of viable KU-19-19 and 647-V cells following treatment with 100 kBq/mL of [^212^Pb]Pb-TCMC-chOI-1 for 24 h. Cells were harvested 24 h post-treatment and stained with FVD-eFluor450 and propidium iodide. Control refers to cells left in the cell medium. **B**) Representative DNA histograms showing untreated 647-V cells (upper) and cells treated with 100 kBq/mL of [^212^Pb]Pb-TCMC-chOI-1 (lower) 24 h post-treatment. Samples were analyzed using a CytoFlex S Flow Cytometer and CytExpert software. Cell cycle distributions are expressed as mean ± SD from three independent experiments performed in triplicate. P-values were calculated using an unpaired t-test with correction for multiple comparisons using the Holm-Sidak method (**p < 0.005, ***p < 0.0005)

Cell cycle analysis on day 1 revealed a significant (p < 0.001) increase in the proportion of cells in the G2/M phase following [^212^Pb]Pb-TCMC-chOI-1 treatment compared to untreated controls, accompanied by a marked reduction in the S phase population in both KU-19-19 and 647-V cell lines (Fig. 5). G2/M arrest was more pronounced in 647-V cells, with up to 76% of cells in G2/M, compared to 58% in KU-19-19.

For the treated cells, G2/M accumulation persisted on days 3 and 6, but with a gradual decrease over time in both cell lines. In KU-19-19, the G2/M fraction decreased from 58% on day 1 to 24% on day 6, with a corresponding recovery of the G1 population. In 647-V cells, G2/M arrest remained substantial on days 3 (40%) and 6 (34%), indicating a more sustained cell cycle blockade. The S phase fraction, which was initially suppressed after treatment, showed partial recovery by day 6 in both cell lines.

## Discussion

This study is the first to demonstrate that PTK7-TAT, via [^212^Pb]Pb-TCMC-chOI-1, elicits potent antigen-specific cytotoxicity in both 2D and 3D BC models. We established a direct relationship between PTK7 surface density, binding kinetics, and therapeutic response, revealing that 3D architecture amplifies the benefits of high-LET alpha emitters compared with conventional 2D models.

The choice of ^212^Pb over alternative alpha-emitters offers several advantages in bladder cancer therapy. Its 10.6 h half-life provides an optimal balance between the very short half-lives of ^213^Bi (45 min) and ^211^At (7.2 h) and the much longer half-life of ^225^Ac (9.9 days) ^223^Ra (11.4 days) (Jabbar et al. 2023). This intermediate half-life allows sufficient time for radiopharmaceutical synthesis and quality control while being appropriate for intravesical instillation (1–4 h). In contrast, for longer-lived radionuclides such as ^225^Ac and ^223^Ra, this short bladder retention period would result in only a small fraction of their total decay occurring during treatment, leaving most of the activity to decay after voiding and potentially increasing systemic exposure. Generator-based production of ^212^Pb from ^228^Th enables a decentralized, on-demand supply comparable to ^213^Bi production from ^225^Ac/^213^Bi generators, but with greater availability and simpler logistics than the cyclotron-dependent production required for ^211^At (Coll et al. 2023; Kokov et al. 2022). Unlike ^223^Ra, which cannot be chelated and has inherent bone-seeking properties that make it unsuitable for cell-specific targeting, ^212^Pb can be conjugated to tumor-targeting vectors for selective delivery (Coll et al. 2023). Additionally, ^212^Pb acts as an in vivo generator for alpha-emitting daughter ^212^Bi, delivering multiple alpha particles per decay while maintaining a simpler decay chain than ^225^Ac, potentially reducing off-target toxicity (Jabbar et al. 2023). The established chelation chemistry and clinical experience with ^212^Pb-labeled compounds further support its suitability for rapid clinical translation (Baidoo et al. 2013). A shorter physical half-life also reduces radiation protection requirements for healthcare workers and simplifies waste disposal considerations.

PTK7 surface densities in tested BC cell lines ranged from ~ 10000 to 70000 sites per cell, levels sufficient for effective radioimmunotherapy based on established thresholds for antigen-mediated targeting (He et al. 2011; Rudnick and Adams 2009; Larson et al 2015). The measured K_D_^APP^ of 10–44 nM matches the affinity previously reported for the same chOI-1 antibody in an ovarian cancer model, showing that chOI-1 retains comparable PTK7 binding across both ovarian and bladder cancer cells (Lindland et al. 2025). Although high-affinity antibodies can enhance initial binding, they may paradoxically impair tumor penetration because of rapid perivascular retention and binding-site barrier effects (Bordeau et al. 2021). Notably, moderate-affinity antibodies, such as chOI-1, can penetrate tumors more uniformly, achieving a deeper tissue distribution than high-affinity variants (He et al. 2011; Rudnick and Adams 2009; Larson et al 2015). This is particularly important for solid tumors with compact architectures or variable target expression, such as BC (Juweid et al 1992; Adams et al. 2001).

The marked difference in cytotoxicity between high (50 MBq/mg) and low (5 MBq/mg) specific activity formulations reflects the fundamental principles of RIC optimization and can be quantitatively explained by antibody-to-radionuclide ratios (Schaffland et al. 2004). For example, for 100 kBq ^212^Pb (containing 5.5 × 10⁹ ^212^Pb atoms), the corresponding number of mAbs (~ 150 kDa) per bound ^212^Pb atom is dramatically different between formulations: 1458 antibodies per ^212^Pb atom for the 50 MBq/mg preparation versus 14585 antibodies per ^212^Pb atom for the 5 MBq/mg formulation. This tenfold increase in “cold” (non-radiolabeled) antibodies in the low-specific activity preparation creates competitive inhibition effects that directly compromise therapeutic efficacy (Larsen et al. 1994). Excess unlabeled antibodies compete for the same PTK7 binding sites as radiolabeled RICs, effectively diluting the radioactive payload delivered to target cells. Higher specific activity formulations minimize the burden of non-therapeutic “cold” antibody mass competing for target sites, thereby optimizing the delivery of radioactive payload per successful binding. This mechanistic advantage is consistent with our results, which show reduced efficacy for both lower-specific-activity formulations and the “cold” chOI-1 pre-blocked samples. At prolonged exposures, both low specific activity [^212^Pb]Pb-TCMC-chOI-1 and the isotype control ([^212^Pb]Pb-TCMC-hIgG at 50 MBq/mg) exhibited measurable effects (4–24 h), likely due to non-specific radiation mechanisms such as crossfire or bystander effects (Pouget and Constanzo 2021; Pouget et al. 2022; Guerra Liberal et al. 2020). Nevertheless, [^212^Pb]Pb-TCMC-chOI-1 consistently outperformed the isotype control, emphasizing the importance of PTK7-specific targeting in achieving superior therapeutic outcomes.

The therapeutic efficacy of TAT is governed by the number of RICs bound to and internalized by a cell and the physical dimensions of the target (Carlin 2018; Lee 2022). Subcellular dosimetry modeling has demonstrated that the absorbed alpha activity increases with tumor radius until it reaches an asymptote near the maximal alpha-particle range of approximately 85 μm (Lee 2022). In 2D monolayers, individual BC cells measure 15–20 μm in diameter, resulting in substantial energy loss as alpha-particle tracks escape into the surrounding medium (Lee 2022). Conversely, our 3D spheroids (~ 200 μm in diameter at the start of the study) captured most alpha tracks and, combined with crossfire effects, markedly increased the average radioactivity deposited within the tumor mass. The internalization of ^212^Pb-RICs further sharpens radioactivity localization and improves the retention of short-lived ^212^Bi daughter (Yong and Brechbiel 2011).

Spheroid models demonstrated markedly enhanced sensitivity compared to 2D monolayers, with IC_50_ values approximately 50-fold lower in 3D systems. This enhanced efficacy reflects the superior geometry of alpha-particle energy absorption and crossfire effects in multicellular structures (Pouget et al. 2022; Nath and Devi 2016). The uniform growth arrest achieved in KU-19-19 spheroids with brief treatment exposures indicates efficient radiation distribution throughout the tumor mass, highlighting the clinical relevance of 3D evaluation systems for the development of TAT. Our integrated analysis revealed that the cell surface binding of [^212^Pb]Pb-TCMC-chOI-1 was a key determinant of cytotoxic efficacy in PTK7-expressing BC models (Table S3). This is supported by the statistically significant differences in bound radioactivity between cell lines and its strong correlation with antigen density and the administered radioactivity required to produce a therapeutic effect. These results emphasize that optimizing antibody binding and antigen targeting is essential to maximize the therapeutic impact of TAT. Although internalized activity did not vary significantly between cell lines and its correlations with efficacy-related variables were not statistically significant, it would be incorrect to dismiss its importance. Internalized alpha-emitting radionuclides are known to deliver higher absorbed radioactivity to the nucleus than surface-bound or extracellular radionuclides (Yong and Brechbiel 2011). In our dataset, internalization was not experimentally blocked; thus, internalized and bound components are not independent, and the strong correlation between bound activity and efficacy likely reflects the combined contribution of both internalized and membrane-bound decays.

Flow cytometry analysis confirmed that [^212^Pb]Pb-TCMC-chOI-1 induced the characteristic hallmarks of high-LET radiation damage, including DNA double-strand breaks, G_2_/M cell cycle arrest, and apoptosis (Pouget and Constanzo 2021; Van den Abbeele et al 1991). The sustained twofold increase in γH2AX levels throughout the observation period indicates persistent complex DNA damage, which is characteristic of alpha-particle exposure (Pouget et al. 2022). The temporal pattern of G2-M arrest, prominent on day 1 but returning to control levels by days 3–6, suggests activation of DNA damage checkpoint recovery mechanisms despite sustained γH2AX elevation (Deckbar et al. 2011). This apparent paradox may reflect cells attempting to progress through compromised checkpoints or the presence of sublethal damage that triggers checkpoint adaptation, rather than permanentarrest (Pouget and Constanzo 2021; Deckbar et al. 2011). The persistence of DNA damage markers alongside checkpoint recovery indicates ongoing genomic instability, which may ultimately lead to a delayed cell death (Pouget and Constanzo 2021). The differential kinetics observed between the cell lines (rapid apoptosis in KU-19-19 cells versus a delayed response in 647-V cells) correlated with their respective PTK7 expression levels and internalization efficiencies.

Several limitations must be considered for future development. Although moderate IRF (12–26%) and antigen expression levels are sufficient for regional therapy applications, they fall below the optimal benchmarks for systemic RIC delivery (Larson et al. 2015). However, regional intravesical administration can concentrate the activity at the tumor site while leveraging crossfire effects for comprehensive tumor coverage (Coll et al. 2023). Importantly, while spheroid growth was effectively inhibited, fluorescence imaging revealed that many cells remained viable despite the treatment. This cytostatic, rather than cytotoxic, effect represents a potential clinical limitation, as viable cells can resume proliferation once radioactive decay is complete. This highlights the need for combination approaches or optimized dosing regimens to achieve complete cell eradication. Future optimization should prioritize achieving higher specific activity through improved chelator-to-antibody ratios, milder labeling conditions, and refined purification methods to enhance immunoreactivity (Van den Abbeele et al. 1991).

For the intravesical delivery of [^212^Pb]Pb-TCMC-chOI-1 in NMIBC, the instillation volume and administered activity should be calibrated to ensure uniform urothelial coverage while preserving local safety and tolerability. Based on clinical precedents in intravesical chemotherapy and immunotherapy, a volume of 20–40 mL is recommended to allow for uniform distribution across the bladder mucosa without over-distention or leakage (Manoharan 2011). Regarding activity, a range of 10–40 MBq [^212^Pb]Pb-TCMC-chOI-1 per instillation has been proposed (Yong and Brechbiel 2015). This corresponds to activity concentrations between 250 and 1000 kBq/mL, depending on the volume instilled. This range is expected to provide therapeutic nuclear hits within the urothelium while maintaining a safety margin. The short half-life of ^212^Pb is compatible with a 1–2 h dwell time, consistent with standard clinical protocols (Seidl 2020; Rousseau et al. 2025). In this study, we used clinically relevant activity concentrations (up to 100 kBq/mL) and incubation times ranging from 1 to 4 h to reflect practical conditions for local bladder cancer therapy.

## Conclusion

This study demonstrates that PTK7-TAT using [^212^Pb]Pb-TCMC-chOI-1 represents a promising therapeutic approach for bladder cancer treatment. The pronounced cytotoxic efficacy observed in three-dimensional spheroid models, with IC_50_ values up to 50-fold lower than two-dimensional cultures, validates the clinical relevance of multicellular evaluation platforms for alpha-particle therapeutics.

The direct correlation between PTK7 expression and therapeutic response, combined with the enhanced crossfire effects in tumor-like architectures, supports the advancement of PTK7-directed therapy for clinical applications. These findings provide compelling preclinical evidence for the development of bladder-sparing intravesical treatments and establish a foundation for future in vivo studies of this novel radiopharmaceutical approach.

## Supplementary Information

Additional file 1.

## Data Availability

The datasets supporting the conclusions of this article are included within the article (and its additional files).

## Abbreviations

2D: Two-dimensional
3D: Three-dimensional
ADC: Antibody–drug conjugate
BC: Bladder cancer
BCG: Bacillus Calmette-Guérin
Bq: Becquerel
DMEM: Dulbecco’s Modified Eagle Medium
DNA: Deoxyribonucleic acid
DSB: Double-strand break
EGFR: Epidermal growth factor receptor
FBS: Fetal bovine serum
FITC: Fluorescein isothiocyanate
IC_50_: Half-maximal inhibitory concentration
IRF: Immunoreactive fraction
IP: Intraperitoneal
K_D_^APP^: Apparent equilibrium dissociation constant
kBq: Kilobecquerel
LET: Linear energy transfer
mAb: Monoclonal antibody
MBq: Megabecquerel
NMIBC: Non-muscle-invasive bladder cancer
PBS: Phosphate-buffered saline
PI: Propidium iodide
PTK7: Protein tyrosine kinase 7
RIC: Radioimmunoconjugate
TAT: Targeted alpha therapy
