# Supplementary material for: Cytotoxicity of 212Pb-labeled anti-PTK7 antibody in 2D adherent and 3D multicellular bladder cancer models
Source: EJNMMI Radiopharm Chem. 2025 Aug 30;10:58. doi: 10.1186/s41181-025-00382-3 (PMC12398452; doi:10.1186/s41181-025-00382-3)
Supplement: Supplementary file 1 — Additional file 1. [file 41181_2025_382_MOESM1_ESM.pptx]

## Slide 1
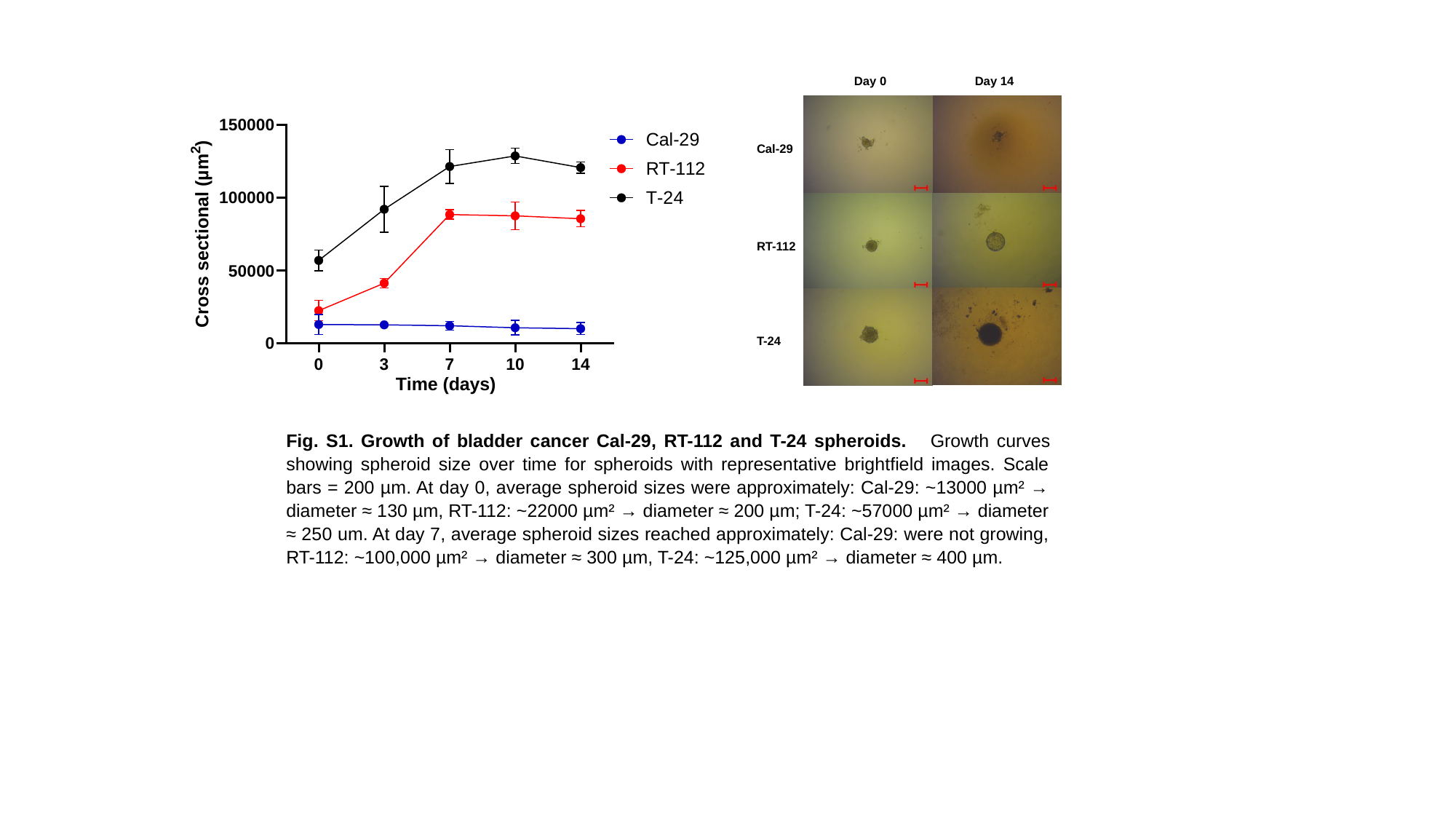

Day 0
Day 14
Cal-29
RT-112
T-24
Fig. S1. Growth of bladder cancer Cal-29, RT-112 and T-24 spheroids. Growth curves showing spheroid size over time for spheroids with representative brightfield images. Scale bars = 200 µm. At day 0, average spheroid sizes were approximately: Cal-29: ~13000 µm² → diameter ≈ 130 µm, RT-112: ~22000 µm² → diameter ≈ 200 µm; T-24: ~57000 µm² → diameter ≈ 250 um. At day 7, average spheroid sizes reached approximately: Cal-29: were not growing, RT-112: ~100,000 µm² → diameter ≈ 300 µm, T-24: ~125,000 µm² → diameter ≈ 400 µm.

## Slide 2
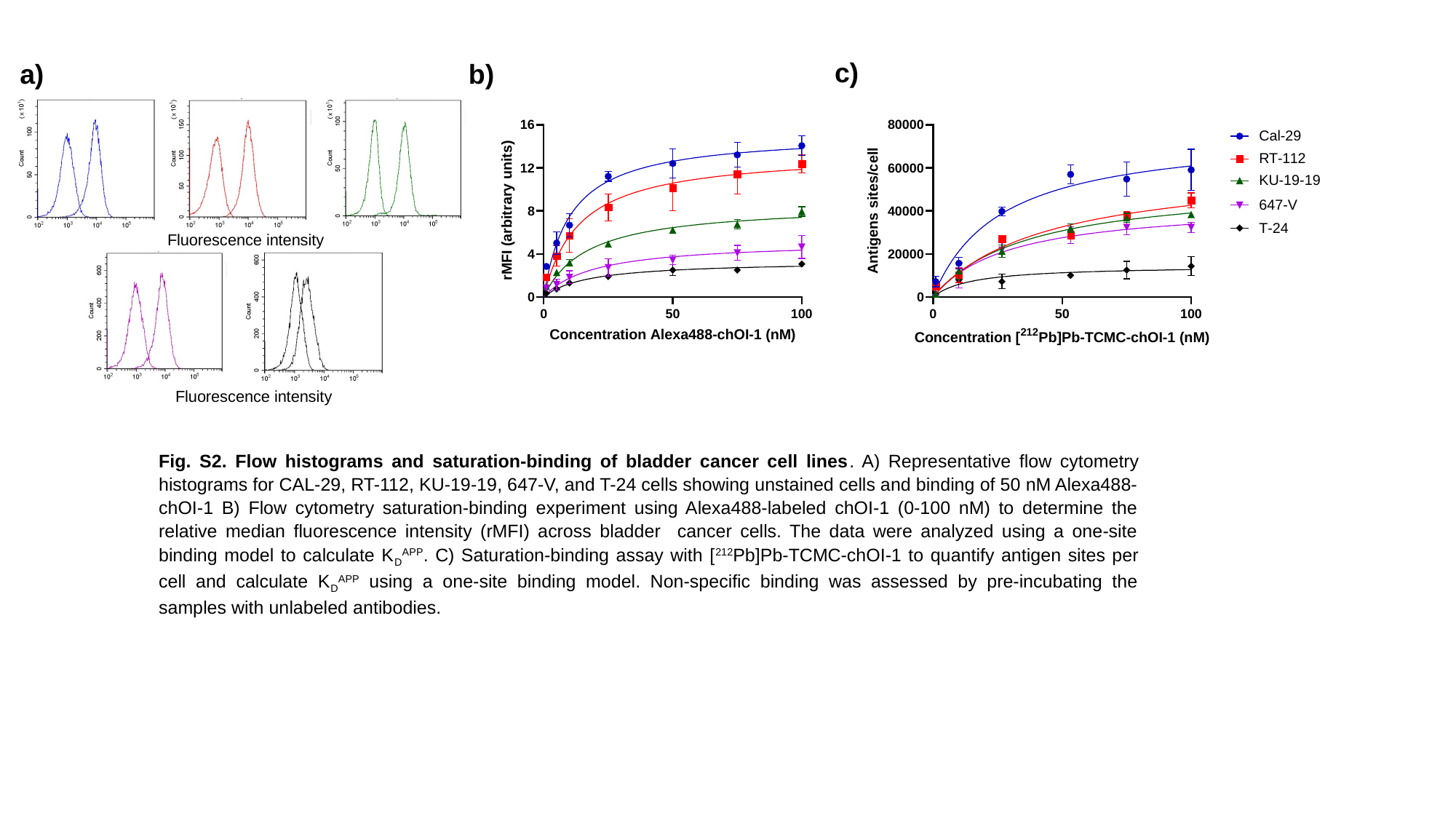

c)
a)
b)
Fluorescence intensity
Fluorescence intensity
Fig. S2. Flow histograms and saturation-binding of bladder cancer cell lines. A) Representative flow cytometry histograms for CAL-29, RT-112, KU-19-19, 647-V, and T-24 cells showing unstained cells and binding of 50 nM Alexa488-chOI-1 B) Flow cytometry saturation-binding experiment using Alexa488-labeled chOI-1 (0-100 nM) to determine the relative median fluorescence intensity (rMFI) across bladder cancer cells. The data were analyzed using a one-site binding model to calculate KDAPP. C) Saturation-binding assay with [212Pb]Pb-TCMC-chOI-1 to quantify antigen sites per cell and calculate KDAPP using a one-site binding model. Non-specific binding was assessed by pre-incubating the samples with unlabeled antibodies.

## Slide 3
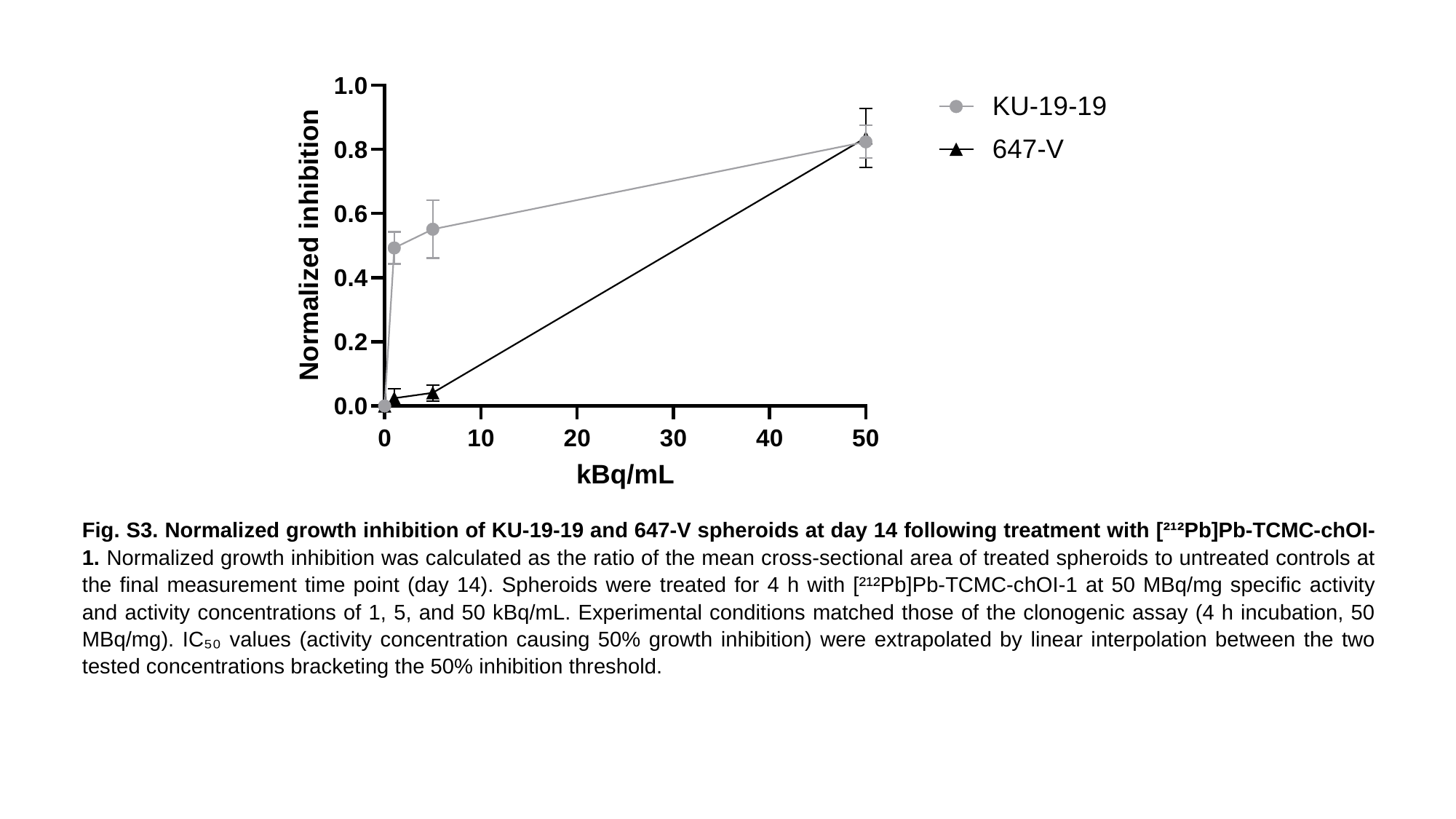

Fig. S3. Normalized growth inhibition of KU-19-19 and 647-V spheroids at day 14 following treatment with [²¹²Pb]Pb-TCMC-chOI-1. Normalized growth inhibition was calculated as the ratio of the mean cross-sectional area of treated spheroids to untreated controls at the final measurement time point (day 14). Spheroids were treated for 4 h with [²¹²Pb]Pb-TCMC-chOI-1 at 50 MBq/mg specific activity and activity concentrations of 1, 5, and 50 kBq/mL. Experimental conditions matched those of the clonogenic assay (4 h incubation, 50 MBq/mg). IC₅₀ values (activity concentration causing 50% growth inhibition) were extrapolated by linear interpolation between the two tested concentrations bracketing the 50% inhibition threshold.

## Slide 4
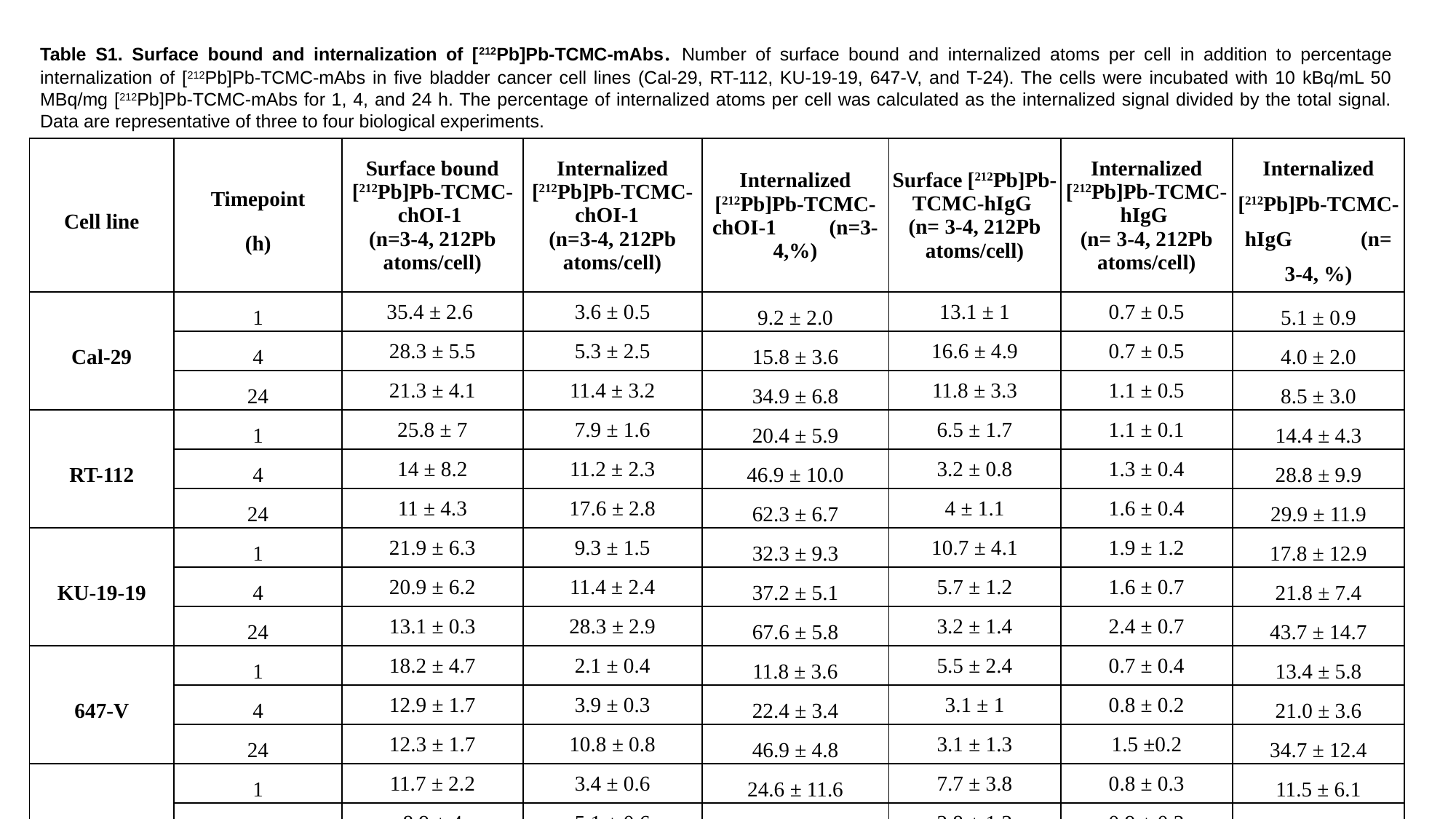

Table S1. Surface bound and internalization of [212Pb]Pb-TCMC-mAbs. Number of surface bound and internalized atoms per cell in addition to percentage internalization of [212Pb]Pb-TCMC-mAbs in five bladder cancer cell lines (Cal-29, RT-112, KU-19-19, 647-V, and T-24). The cells were incubated with 10 kBq/mL 50 MBq/mg [212Pb]Pb-TCMC-mAbs for 1, 4, and 24 h. The percentage of internalized atoms per cell was calculated as the internalized signal divided by the total signal. Data are representative of three to four biological experiments.
| Cell line | Timepoint (h) | Surface bound [212Pb]Pb-TCMC-chOI-1 (n=3-4, 212Pb atoms/cell) | Internalized [212Pb]Pb-TCMC-chOI-1 (n=3-4, 212Pb atoms/cell) | Internalized [212Pb]Pb-TCMC-chOI-1 (n=3-4,%) | Surface [212Pb]Pb-TCMC-hIgG (n= 3-4, 212Pb atoms/cell) | Internalized [212Pb]Pb-TCMC-hIgG (n= 3-4, 212Pb atoms/cell) | Internalized [212Pb]Pb-TCMC-hIgG (n= 3-4, %) |
| --- | --- | --- | --- | --- | --- | --- | --- |
| Cal-29 | 1 | 35.4 ± 2.6 | 3.6 ± 0.5 | 9.2 ± 2.0 | 13.1 ± 1 | 0.7 ± 0.5 | 5.1 ± 0.9 |
| | 4 | 28.3 ± 5.5 | 5.3 ± 2.5 | 15.8 ± 3.6 | 16.6 ± 4.9 | 0.7 ± 0.5 | 4.0 ± 2.0 |
| | 24 | 21.3 ± 4.1 | 11.4 ± 3.2 | 34.9 ± 6.8 | 11.8 ± 3.3 | 1.1 ± 0.5 | 8.5 ± 3.0 |
| RT-112 | 1 | 25.8 ± 7 | 7.9 ± 1.6 | 20.4 ± 5.9 | 6.5 ± 1.7 | 1.1 ± 0.1 | 14.4 ± 4.3 |
| | 4 | 14 ± 8.2 | 11.2 ± 2.3 | 46.9 ± 10.0 | 3.2 ± 0.8 | 1.3 ± 0.4 | 28.8 ± 9.9 |
| | 24 | 11 ± 4.3 | 17.6 ± 2.8 | 62.3 ± 6.7 | 4 ± 1.1 | 1.6 ± 0.4 | 29.9 ± 11.9 |
| KU-19-19 | 1 | 21.9 ± 6.3 | 9.3 ± 1.5 | 32.3 ± 9.3 | 10.7 ± 4.1 | 1.9 ± 1.2 | 17.8 ± 12.9 |
| | 4 | 20.9 ± 6.2 | 11.4 ± 2.4 | 37.2 ± 5.1 | 5.7 ± 1.2 | 1.6 ± 0.7 | 21.8 ± 7.4 |
| | 24 | 13.1 ± 0.3 | 28.3 ± 2.9 | 67.6 ± 5.8 | 3.2 ± 1.4 | 2.4 ± 0.7 | 43.7 ± 14.7 |
| 647-V | 1 | 18.2 ± 4.7 | 2.1 ± 0.4 | 11.8 ± 3.6 | 5.5 ± 2.4 | 0.7 ± 0.4 | 13.4 ± 5.8 |
| | 4 | 12.9 ± 1.7 | 3.9 ± 0.3 | 22.4 ± 3.4 | 3.1 ± 1 | 0.8 ± 0.2 | 21.0 ± 3.6 |
| | 24 | 12.3 ± 1.7 | 10.8 ± 0.8 | 46.9 ± 4.8 | 3.1 ± 1.3 | 1.5 ±0.2 | 34.7 ± 12.4 |
| T-24 | 1 | 11.7 ± 2.2 | 3.4 ± 0.6 | 24.6 ± 11.6 | 7.7 ± 3.8 | 0.8 ± 0.3 | 11.5 ± 6.1 |
| | 4 | 8.9 ± 4 | 5.1 ± 0.6 | 37.7 ± 7.1 | 2.8 ± 1.3 | 0.9 ± 0.3 | 24.1 ± 1.5 |
| | 24 | 10.8 ± 2.6 | 13.6 ± 2.7 | 55.8 ± 1.9 | 3 ± 1.3 | 3.2 ± 0.6 | 45.8 ± 8.4 |

## Slide 5
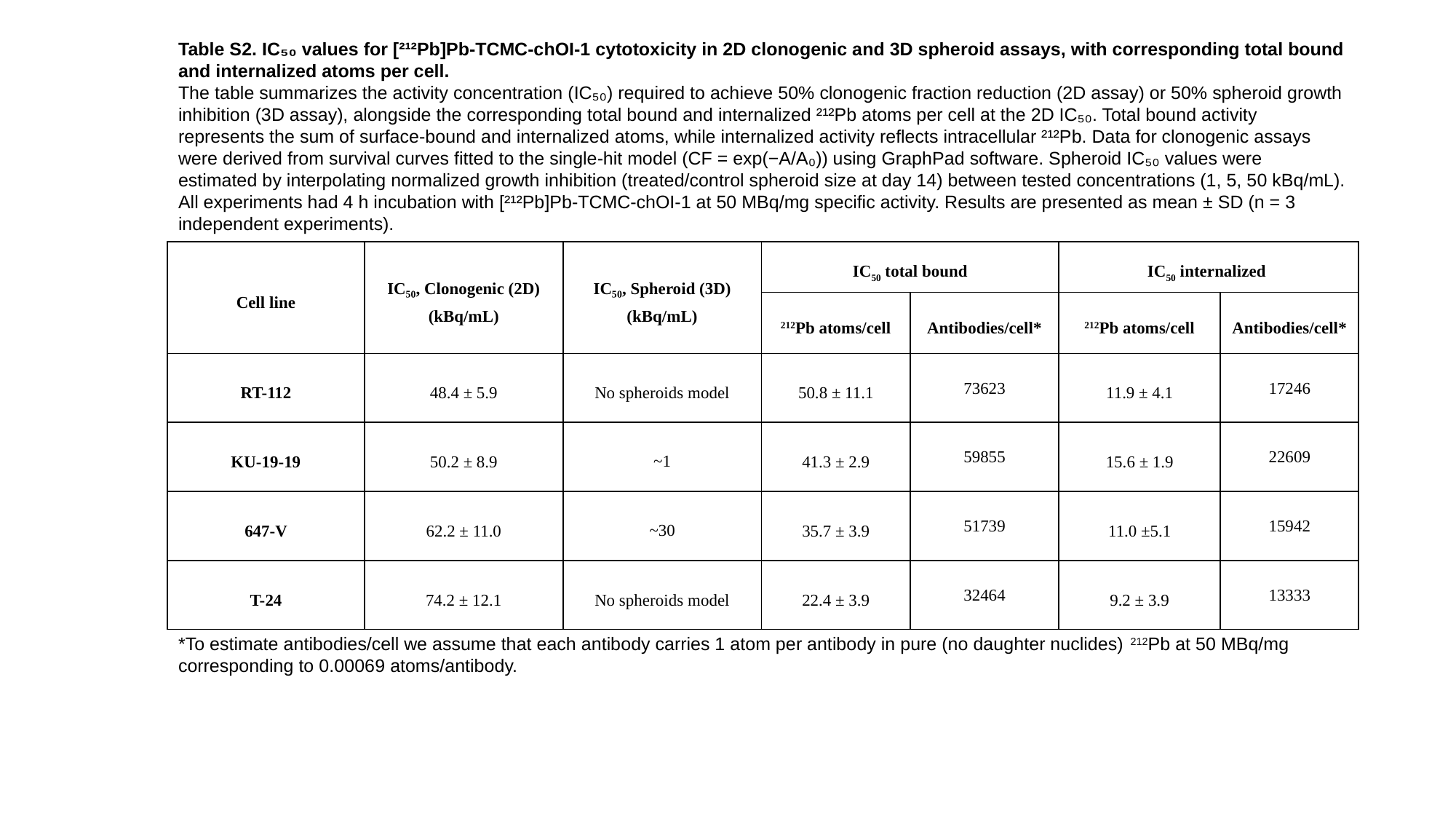

Table S2. IC₅₀ values for [²¹²Pb]Pb-TCMC-chOI-1 cytotoxicity in 2D clonogenic and 3D spheroid assays, with corresponding total bound and internalized atoms per cell.
The table summarizes the activity concentration (IC₅₀) required to achieve 50% clonogenic fraction reduction (2D assay) or 50% spheroid growth inhibition (3D assay), alongside the corresponding total bound and internalized ²¹²Pb atoms per cell at the 2D IC₅₀. Total bound activity represents the sum of surface-bound and internalized atoms, while internalized activity reflects intracellular ²¹²Pb. Data for clonogenic assays were derived from survival curves fitted to the single-hit model (CF = exp(−A/A₀)) using GraphPad software. Spheroid IC₅₀ values were estimated by interpolating normalized growth inhibition (treated/control spheroid size at day 14) between tested concentrations (1, 5, 50 kBq/mL). All experiments had 4 h incubation with [²¹²Pb]Pb-TCMC-chOI-1 at 50 MBq/mg specific activity. Results are presented as mean ± SD (n = 3 independent experiments).
| Cell line | IC₅₀, Clonogenic (2D) (kBq/mL) | IC₅₀, Spheroid (3D) (kBq/mL) | IC50 total bound | | IC50 internalized | |
| --- | --- | --- | --- | --- | --- | --- |
| | | | 212Pb atoms/cell | Antibodies/cell\* | 212Pb atoms/cell | Antibodies/cell\* |
| RT-112 | 48.4 ± 5.9 | No spheroids model | 50.8 ± 11.1 | 73623 | 11.9 ± 4.1 | 17246 |
| KU-19-19 | 50.2 ± 8.9 | ~1 | 41.3 ± 2.9 | 59855 | 15.6 ± 1.9 | 22609 |
| 647-V | 62.2 ± 11.0 | ~30 | 35.7 ± 3.9 | 51739 | 11.0 ±5.1 | 15942 |
| T-24 | 74.2 ± 12.1 | No spheroids model | 22.4 ± 3.9 | 32464 | 9.2 ± 3.9 | 13333 |
*To estimate antibodies/cell we assume that each antibody carries 1 atom per antibody in pure (no daughter nuclides) 212Pb at 50 MBq/mg corresponding to 0.00069 atoms/antibody.

## Slide 6
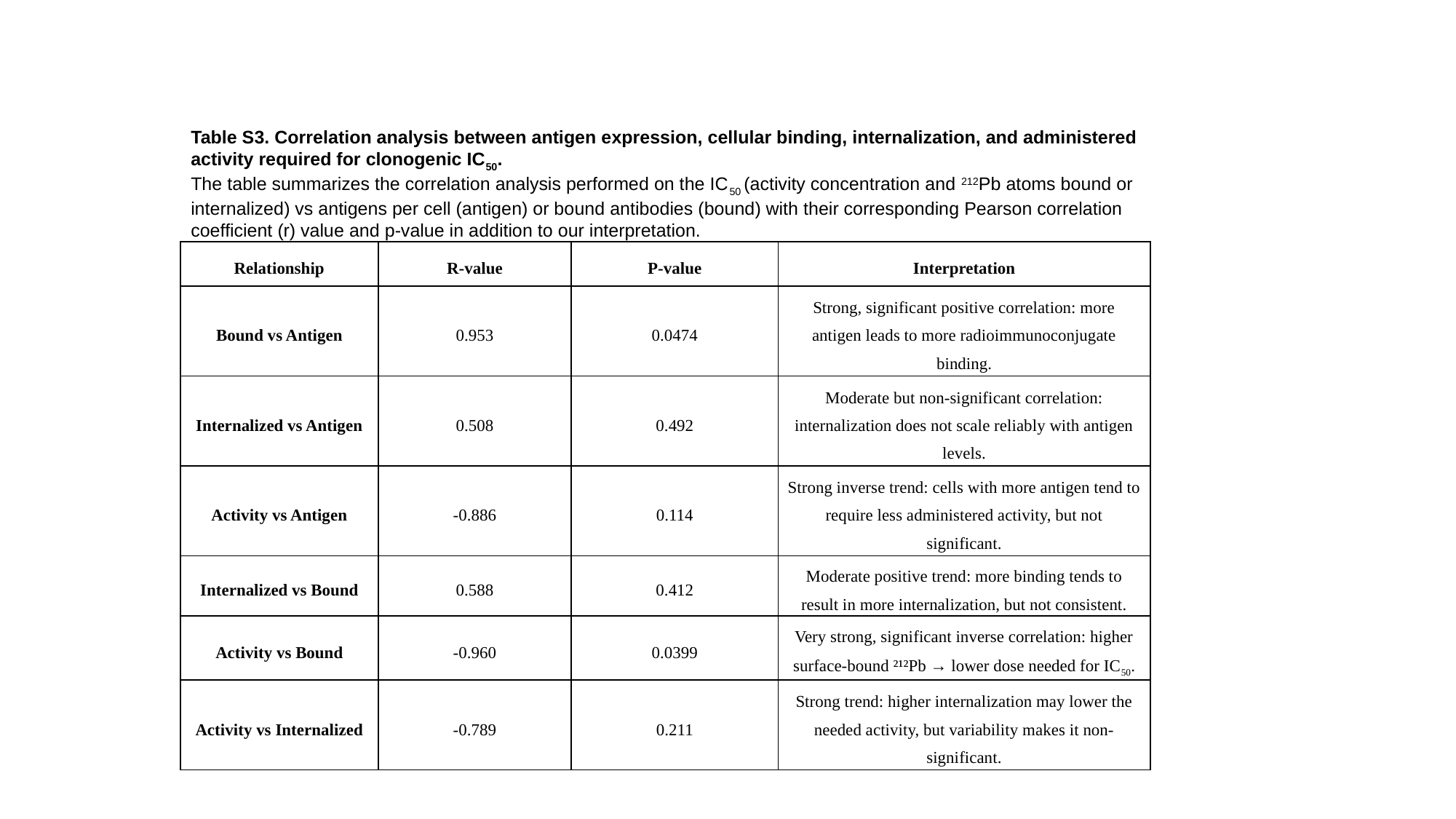

Table S3. Correlation analysis between antigen expression, cellular binding, internalization, and administered activity required for clonogenic IC50.
The table summarizes the correlation analysis performed on the IC50 (activity concentration and 212Pb atoms bound or internalized) vs antigens per cell (antigen) or bound antibodies (bound) with their corresponding Pearson correlation coefficient (r) value and p-value in addition to our interpretation.
| Relationship | R-value | P-value | Interpretation |
| --- | --- | --- | --- |
| Bound vs Antigen | 0.953 | 0.0474 | Strong, significant positive correlation: more antigen leads to more radioimmunoconjugate binding. |
| Internalized vs Antigen | 0.508 | 0.492 | Moderate but non-significant correlation: internalization does not scale reliably with antigen levels. |
| Activity vs Antigen | -0.886 | 0.114 | Strong inverse trend: cells with more antigen tend to require less administered activity, but not significant. |
| Internalized vs Bound | 0.588 | 0.412 | Moderate positive trend: more binding tends to result in more internalization, but not consistent. |
| Activity vs Bound | -0.960 | 0.0399 | Very strong, significant inverse correlation: higher surface-bound ²¹²Pb → lower dose needed for IC50. |
| Activity vs Internalized | -0.789 | 0.211 | Strong trend: higher internalization may lower the needed activity, but variability makes it non-significant. |

## Slide 7
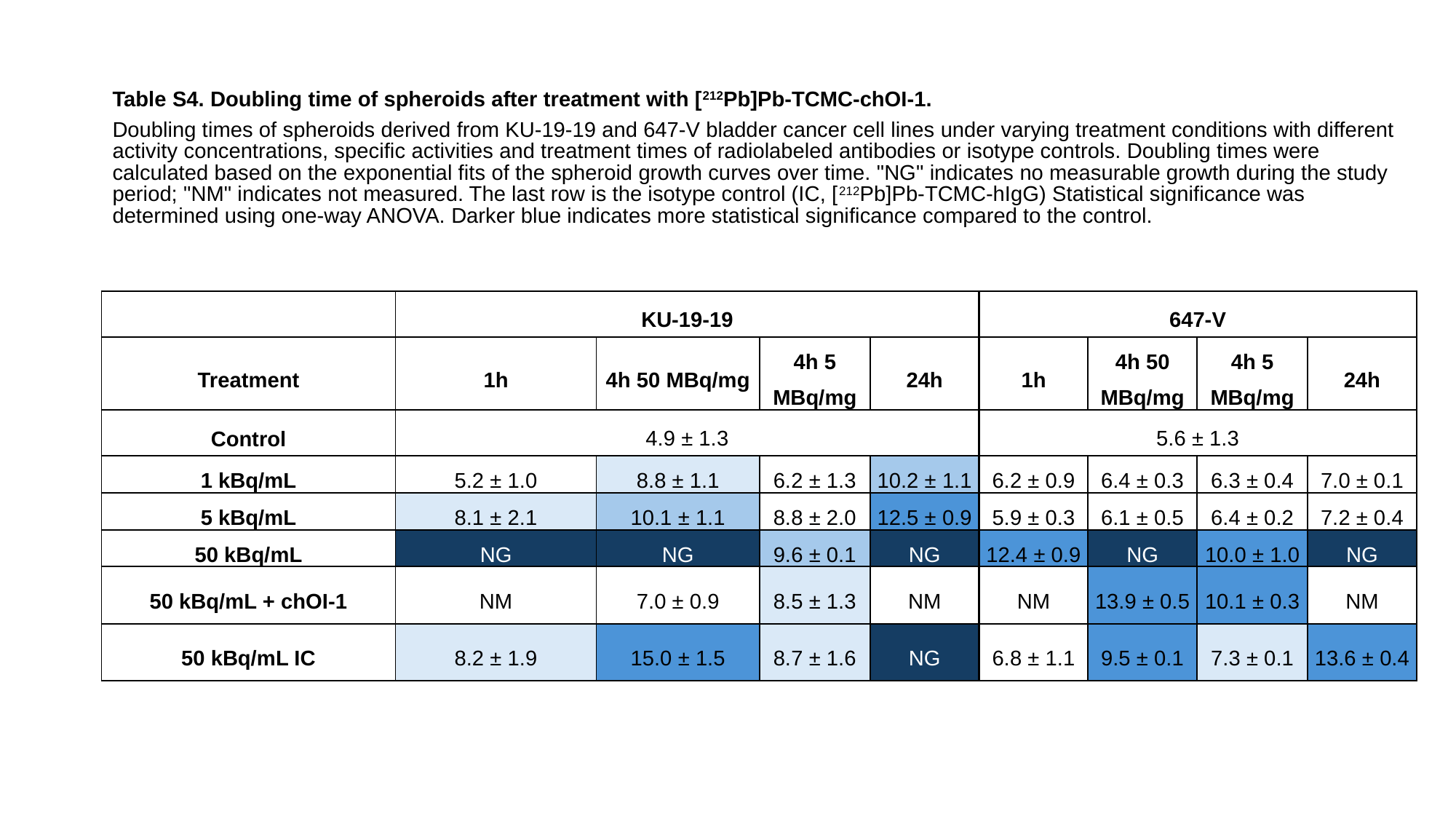

Table S4. Doubling time of spheroids after treatment with [212Pb]Pb-TCMC-chOI-1.
Doubling times of spheroids derived from KU-19-19 and 647-V bladder cancer cell lines under varying treatment conditions with different activity concentrations, specific activities and treatment times of radiolabeled antibodies or isotype controls. Doubling times were calculated based on the exponential fits of the spheroid growth curves over time. "NG" indicates no measurable growth during the study period; "NM" indicates not measured. The last row is the isotype control (IC, [212Pb]Pb-TCMC-hIgG) Statistical significance was determined using one-way ANOVA. Darker blue indicates more statistical significance compared to the control.
| | KU-19-19 | | | | 647-V | | | |
| --- | --- | --- | --- | --- | --- | --- | --- | --- |
| Treatment | 1h | 4h 50 MBq/mg | 4h 5 MBq/mg | 24h | 1h | 4h 50 MBq/mg | 4h 5 MBq/mg | 24h |
| Control | 4.9 ± 1.3 | | | | 5.6 ± 1.3 | | | |
| 1 kBq/mL | 5.2 ± 1.0 | 8.8 ± 1.1 | 6.2 ± 1.3 | 10.2 ± 1.1 | 6.2 ± 0.9 | 6.4 ± 0.3 | 6.3 ± 0.4 | 7.0 ± 0.1 |
| 5 kBq/mL | 8.1 ± 2.1 | 10.1 ± 1.1 | 8.8 ± 2.0 | 12.5 ± 0.9 | 5.9 ± 0.3 | 6.1 ± 0.5 | 6.4 ± 0.2 | 7.2 ± 0.4 |
| 50 kBq/mL | NG | NG | 9.6 ± 0.1 | NG | 12.4 ± 0.9 | NG | 10.0 ± 1.0 | NG |
| 50 kBq/mL + chOI-1 | NM | 7.0 ± 0.9 | 8.5 ± 1.3 | NM | NM | 13.9 ± 0.5 | 10.1 ± 0.3 | NM |
| 50 kBq/mL IC | 8.2 ± 1.9 | 15.0 ± 1.5 | 8.7 ± 1.6 | NG | 6.8 ± 1.1 | 9.5 ± 0.1 | 7.3 ± 0.1 | 13.6 ± 0.4 |
